# Activation of Vago by interferon regulatory factor (IRF) suggests an interferon system-like antiviral mechanism in shrimp

**DOI:** 10.1038/srep15078

**Published:** 2015-10-13

**Authors:** Chaozheng Li, Haoyang Li, Yixiao Chen, Yonggui Chen, Sheng Wang, Shao-Ping Weng, Xiaopeng Xu, Jianguo He

**Affiliations:** 1 MOE Key Laboratory of Aquatic Product Safety/State Key Laboratory for Biocontrol, School of Life Sciences, Sun Yat-sen University, Guangzhou, P.R. China; 2 Institute of Aquatic Economic Animals and Guangdong Provice Key Laboratory for Aquatic Economic Animals, Sun Yat-sen University, Guangzhou, P.R. China; 3 School of Marine Sciences, Sun Yat-sen University, Guangzhou, P.R. China; 4 South China Sea Resource Exploitation and Protection Collaborative Innovation Center (SCS-REPIC), Guangzhou, P.R. China

## Abstract

There is a debate on whether invertebrates possess an antiviral immunity similar to the interferon (IFN) system of vertebrates. The Vago gene from arthropods encodes a viral-activated secreted peptide that restricts virus infection through activating the JAK-STAT pathway and is considered to be a cytokine functionally similar to IFN. In this study, the first crustacean IFN regulatory factor (IRF)-like gene was identified in Pacific white shrimp, *Litopenaeus vannamei*. The *L. vannamei* IRF showed similar protein nature to mammalian IRFs and could be activated during virus infection. As a transcriptional regulatory factor, *L. vannamei* IRF could activate the IFN-stimulated response element (ISRE)-containing promoter to regulate the expression of mammalian type I IFNs and initiate an antiviral state in mammalian cells. More importantly, IRF could bind the 5′-untranslated region of *L. vannamei* Vago4 gene and activate its transcription, suggesting that shrimp Vago may be induced in a similar manner to that of IFNs and supporting the opinion that Vago might function as an IFN-like molecule in invertebrates. These suggested that shrimp might possess an IRF-Vago-JAK/STAT regulatory axis, which is similar to the IRF-IFN-JAK/STAT axis of vertebrates, indicating that invertebrates might possess an IFN system-like antiviral mechanism.

In vertebrates, the interferon (IFN) response, characterized by induction of IFNs and the subsequent establishment of the cellular antiviral state, is the hallmark of antiviral immunity. IFNs are a group of secreted cytokines with activities to inhibit viral replication and regulate the function of immune cells^1^,^2^. In mammals, three types of IFNs (type I, II and III IFNs) have been identified, all exhibiting significant antiviral activities^3^,^4^. Activation of type I and III IFNs, occurring in various cells in response to viral infection, is considered to be central to the antiviral innate immunity in vertebrates^3^,^5^,^6^. The IFN regulatory factor (IRF) family is a group of transcriptional factors that play critical roles in activation of IFNs^7^,^8^. Up to now, nine IRFs, IRF-1 to -9, have been identified in mammals, all containing a highly conserved DNA-binding domain in the amino-terminal region known to recognize the DNA consensus sequence similar to the IFN-stimulated response element (ISRE)^8^,^9^. The carboxy terminal region of IRFs, more diverse than the amino-terminal region, is responsible for specific transcriptional activities and biological functions by mediating specific interactions between IRFs and other transcription factors or cofactors^10^,^11^.

Among the IRF family, IRF-3 and IRF-7 are essential for the regulated expression of IFNs^8^,^9^. In mammals, IRF-3 is constitutively expressed, while IRF7 is low-expressed in most cells and can be strongly induced by type I IFN via the JAK/STAT pathway and thus itself is an IFN-stimulated gene (ISG)^12^,^13^. On infection of virus, host pattern recognition receptors (PRRs) sense viral pathogen-associated molecular patterns (PAMPs) to initiate immune responses. To date, numerous PRRs that specifically recognize foreign nucleic acids have been identified in mammals, such as Toll-like receptor 3, 8, 9 (TLR3, TLR8, and TLR9), DNA-dependent activator of IRFs (DAI), interferon-gamma-inducible protein 16 (IFI16), RIG-I-like receptors (RLRs) and Leucine-rich repeat flightless-interacting protein 1 (LRRFIP1)^14^,^15^,^16^,^17^,^18^,^19^. These virus-activated PRRs trigger signaling cascades leading to activation of TANK-binding kinase 1 (TBK1) and inhibitor of NF-kB kinase ε (IKK-ε), which in turn phosphorylate IRF-3 and IRF-7^20^,^21^. The phosphorylation mediates the formation of IRF3 homodimers, IRF7 homodimers, or IRF3/IRF7 heterodimers, which translocate into the nucleus to bind the virus responsive element (VRE)/ISRE region within the promoters of IFNs to activate their expression^22^,^23^. The secreted IFNs bind to IFN receptors to activate expression of hundreds of ISGs through the JAK/STAT pathway. These processes lead to the activation of the IFN system and determine the establishment of the antiviral state in vertebrate cells.

The origin and evolution of the IFN system have attracted increasing attention in recent years. Since initially discovered in human cells in the 1950s, multiple homologous subgroups of the IFN family have been identified in vertebrates from fish to mammals^24^. The origin of IFN protein with conserved sequence could be evolutionarily derived from teleosts^25^,^26^. The fish IFN genes show similarities with those of mammals and play important role in antiviral immunity^27^,^28^. Besides, a total of eleven IRF family members have been identified in fish to date, among which IRF-1, -3, and -7 have been evidenced to play vital roles in IFN responses^29^,^30^,^31^. As the IFN homologous gene has not been found in invertebrate genomes so far, it had been thought that the IFN signaling pathway was absent from invertebrates. However, recent studies have suggested that invertebrates possess nucleic acid-induced antiviral immunity, which may be similar to the IFN responses of mammals^32^,^33^,^34^,^35^. The JAK-STAT pathway as well as many ISG-homologous genes and nucleic acid-recognizing PRRs have also been identified in invertebrates and proved to be essential for the antiviral responses^36^,^37^,^38^,^39^. Moreover, a number of IRF-like genes have been explored in genomes and expressed sequence tag (EST) databases of many invertebrates, covering all principal metazoan groups except Nematoda and Hexapoda^40^,^41^,^42^. More importantly, the novel identified Vago gene from arthropods, encoding a viral-activated secreted peptide that restricts virus infection in infected and neighboring cells by activating the JAK-STAT pathway, is considered to be an arthropod cytokine similar to vertebrate IFN in function (not in sequence)^43^,^44^. These offer a new insight into the invertebrate antiviral immunity and lead us reconsider the question whether invertebrates have the IFN system.

Activation of Vago can be induced independent of an RNAi response through recognition of viral double-stranded RNA (dsRNA) by Dicer-2, a DExD/H-box helicase with similarity to vertebrate RIG-I–like receptors^44^. A recent study revealed that the TRAF-Rel2 signaling pathway is involved in the activation of Vago after viral infection^45^. However, the conclusion that Vago is a bona fide functional homolog of interferon needs further support. Moreover, the function of invertebrate IRFs remains largely unknown and the functionally evolutionary relation between invertebrate IRFs and vertebrate IFN pathway is still unclear. In this study, we identified an IRF-like gene from Pacific white shrimp, *Litopenaeus vannamei*, which is the first studied IRF gene in crustaceans. We demonstrated that the *L. vannamei* IRF plays a role in the context of host defense against white spot syndrome virus (WSSV) infection and can activate the IFN response to induce an antiviral state in mammalian cells. Moving forward, we showed that *L. vannamei* IRF can directly bind the Vago promoter to regulate its transcription, which suggests that the regulatory mechanism of Vago induction could be similar to that of IFN in vertebrates, strongly supporting the hypothesis that Vago is an IFN-like molecule in invertebrates. Furthermore, these exhibited that the crustacean immunity could have an IRF-Vago-JAK/STAT pathway regulatory axis that shows similarity to the IRF-IFN-JAK/STAT pathway axis of vertebrates, suggesting that invertebrates might possess an IFN system-like antiviral mechanism.

## Results

### Bioinformatics and expression analysis of *L. vannamei* IRF

The full length of the *L. vannamei* IRF transcript is 1416 bp, comprising a 202 bp 5′-untranslated region (5′-UTR), a 125 bp 3′-UTR and a 1089 bp ORF encoding a 362 amino acids protein (Fig. S1A). The *L. vannamei* IRF has a DNA-binding domain in the amino-terminal regions that is homologous to those of vertebrate IRFs (Fig. S1B). The putative DNA-binding domain of *L. vannamei* IRF exhibits 43%, 43% and 42% similarities to those of fishes *Paralichthys olivaceus*, *Takifugu rubripes*, and *Epinephelus coioides*, respectively, and also shows 24% identity and 45% similarity to human IRF2, suggesting a evolutionary relation between crustacean and vertebrates IRFs.

**Figure 1:**
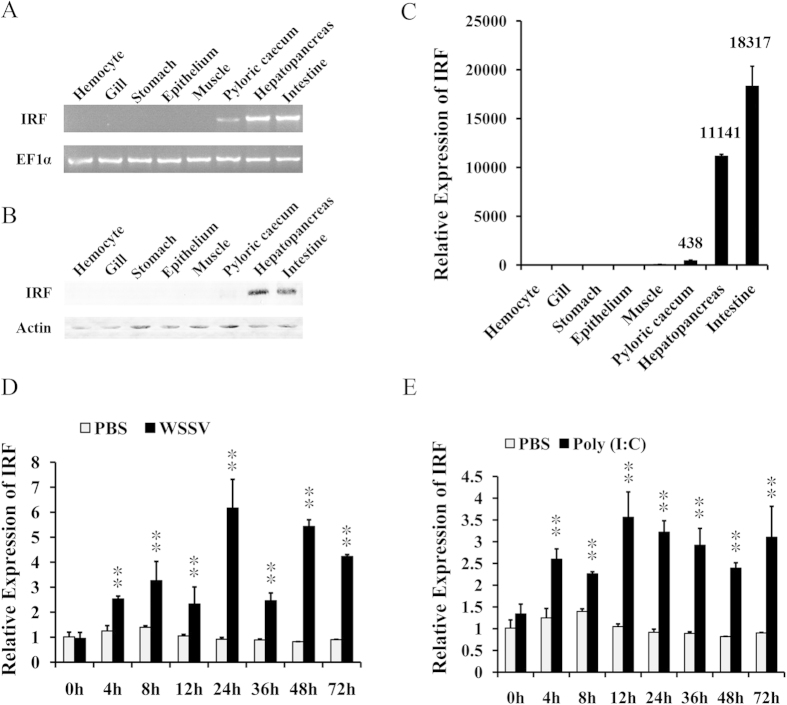
Expression of IRF in *L. vannamei* tissues. Tissue distribution of IRF was detected by RT-PCR (**A**), western-blot (**B**) and real-time PCR (**C**). Real-time RT-PCR was performed in triplicate for each sample using EF-1α gene as internal control by the Livak (2^−△△CT^) method. Expression levels were provided as the mean fold changes (means ± SD, n = 3) relative to that in hemocytes, which was set as 1.0. (**D**,**E**) Expression profiles of IRF after WSSV and poly (I:C) challenges analyzed by real-time PCR. The expression level at 0 h post PBS injection was set as baseline (1.0). **p < 0.01.

The mRNA and protein levels of *L. vannamei* IRF in tissues were analyzed using RT-PCR and western-blot, which provided consistent results. Expression of IRF was detected at high level in hepatopancreas and intestines, weakly in pyloric caecus, but not in other detected tissues (Fig. 1A,B). The results were validated by real-time PCR, which demonstrated that the expression of IRF in intestines, hepatopancreas and pyloric caecus was 18317-, 11141- and 438-fold over that in hemocytes, respectively (Fig. 1C). The expression profile of IRF in hepatopancreas upon immune stimulation was also detected using real-time PCR. After WSSV or poly (I:C) injection, the expression of IRF was significantly up-regulated making a periodical shape of expression curve, suggesting IRF could be activated by virus infection or foreign dsRNA challenge (Fig. 1D,E).

### Dimmerization of IRF

**Figure 2:**
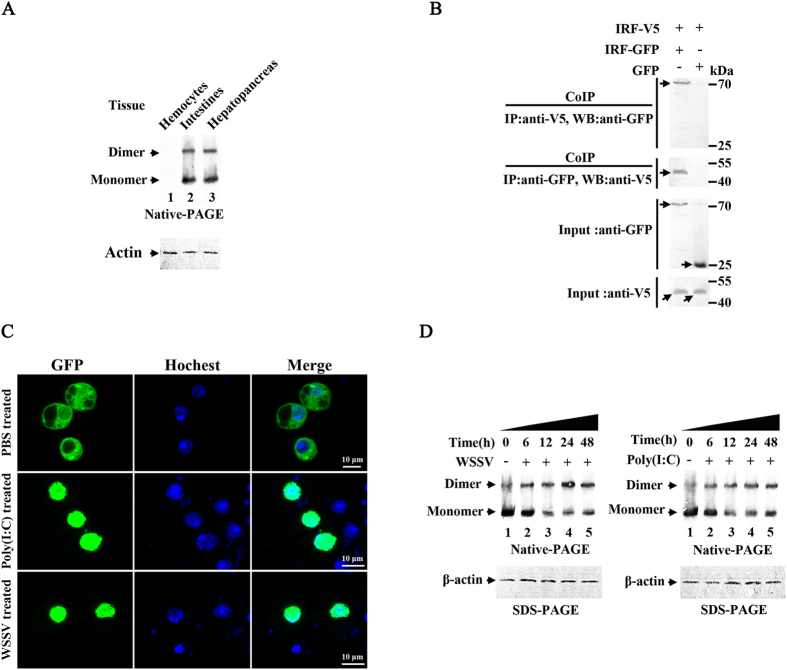
Dimmerization and subcellular localization of *L. vannamei* IRF protein. (**A**) IRF dimmers in intestines, hepatopancreas and hemocytes (as negative control) were analyzed using native PAGE electrophoresis coupled with western-blot.(**B**) Interaction between GFP- and V5-tagged IRF proteins analyzed by co-immunoprecipitation (Co-IP). (**C**) The nuclear translocation of IRF after WSSV or poly (I:C) stimulation. *Drosophila* S2 cells expressing GFP-tagged IRF (green) were treated with Hochest 33258 to counterstain nuclei (blue) and observed under confocal laser scanning microscope. (**D**) Dimmerization of IRF in shrimp hepatopancreas analyzed by native PAGE electrophoresis and western-blot.

We analyzed the IRF protein in shrimp tissues by western-blot using native-PAGE with β-actin as control (Fig. 2A). The bands corresponding to the IRF dimmers and monomers could be observed in intestines and hepatopancreas, but not in the control hemocytes, which had been shown above not to express IRF. The interaction between L. vannamei IRF molecules was further analyzed by co-immunoprecipitation in S2 cells co-expressing V5-tagged and GFP-tagged IRFs (Fig. 2B). The result demonstrated that IRF could interact with itself but not the control GFP protein, confirming that the IRF protein could form dimmers.

Given that WSSV infection and poly (I:C) challenge can up-regulate the expression of IRF, we investigated their activation effects on IRF functions. The subcellular localization of GFP-tagged IRF was detected using confocal laser scanning microscopy (Fig. 2C). In PBS mock treated cells, IRF was mainly present in the cytoplasm, while after WSSV or poly (I:C) treatment, IRF mainly located in the nucleus, suggesting WSSV and poly (I:C) could stimulate the translocation of IRF from the cytoplasm into the nucleus. We further detected the formation of IRF dimmers in WSSV or poly (I:C)-treated cells using native-PAGE and western-blot (Fig. 2D). In both the WSSV and poly (I:C) treated groups, following the prolongation of treatment, more IRF dimmers were formed with the highest ratio of dimmer to monomer observed at 24 h post treatment, suggesting that WSSV or poly (I:C) stimulation could promote the formation of IRF dimmers.

### Transcription factor activity of IRF

**Figure 3:**
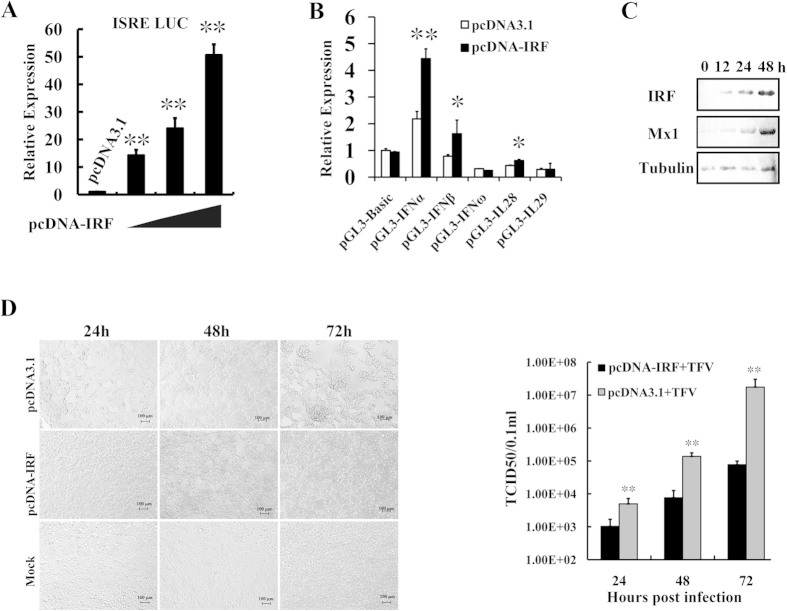
Transcriptional regulatory function and antiviral acitivity of *L. vannamei* IRF. (**A**) Activation of ISRE-containing promoter by IRF detected using dual luciferase reporter assays performed on *Drosophila* S2 cells. (**B**) Transcriptional regulatory effects of *L. vannamei* IRF on mammalian type I and III IFNs in HEK293T cells. (**C**) Activation of Mx1 protein in *L. vannamei* IRF-expressed HEK293T cells detected by western-blot. (**D**) *L. vannamei* IRF triggers an antiviral state in mammalian cells. HEK293T cells were transfected with pcDNA-IRF and pcDNA 3.1 (as control) and infected with Tiger frog virus (TFV). Left panel, pathological signs of TFV-infected cells at 24, 48 and 72 h post infection; right panel, titers of the released TFV in the cell supernatants analyzed using TCID_50_ method.

The *L. vannamei* IRF showed a similarity to vertebrate IRFs and could translocate into the nucleus in response to immune stimulation, indicating *L. vannamei* IRF could function as a transcription factor. As *L. vannamei* IRF contains a DNA-binding domain similar to those of mammalian IRFs, we investigated the effect of *L. vannamei* IRF on a promoter containing ISRE through dual luciferase reporter gene assays (Fig. 3A). The results showed that as the level of the transfected IRF-expressing plasmid increased from 20 to 30 and 50 ng, the activity of the promoter was also up-regulated from 14.3- to 24.0- and 50.6-fold compared with the control, respectively. This suggested that *L. vannamei* IRF could have transcriptional regulation activity on promoters containing mammalian ISRE sequence. As it has been known that promoters of many human type I and III IFNs can be regulated by human IRFs, we investigated the effects of *L. vannamei* IRF on promoters of human type I and III IFNs (Fig. 3B). The *L. vannamei* IRF could significantly enhance the activities of the promoters of type I IFNs, IFN-α and –β, by 2.0- and 2.1-fold compared with the control, respectively, whereas it had no effect on the promoters of type I IFN IFN–ω and type III IFNs IL28 and IL29.

Mx1 is an interferon-induced GTP-binding protein with antiviral activity^46^. Using western-blot, we investigated the expression of Mx1 in *L. vannamei* IRF-expressing cells. After IRF transfection, with the increase of IRF expression, the level of Mx1 was up-regulated, while the internal control tubulin protein remained unchanged (Fig. 3C). This confirmed that *L. vannamei* IRF could function in mammalian cells and be involved in the IFN response.

Tiger frog virus (TFV) is a member of the genus Ranavirus, family Iridoviridae and can infect a wide range of cell lines including mammalian cells^47^,^48^. HEK293T cells were transfected with pcDNA-IRF or original pcDNA3.1 plasmid (as control) and then experimentally infected with TFV. We observed that at 24 h, 48, and 72 h post infection, the cytopathic signs of *L. vannamei* IRF-expressing cells were obviously milder than the control cells (Fig. 3D, left panel). The titers of the released TFV in the cell supernatants were analyzed using TCID_50_ method (Fig. 3D, right panel), which demonstrated that compared with the control, the TFV titers in the supernatants of IRF-expressing cells were significantly decreased at each time point. These suggested that as a transcription factor, *L. vannamei* IRF could trigger an antiviral state in mammalian cells.

### Regulation of Vago promoter by IRF

**Figure 4:**
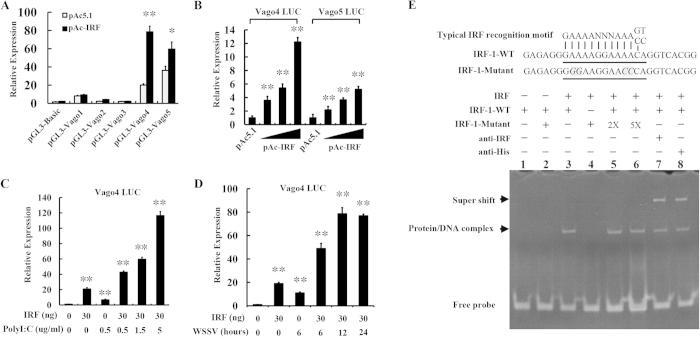
Regulation of *L. vannamei* Vago by IRF. (**A**) Transcriptional regulatory effects of IRF on 5 isoforms of *L. vannamei* Vago investigated by dual luciferase reporter assays. (**B**) Regulation of the activities of Vago4 and Vago5 promoters by improving levels of IRF expression. (**C**,**D**) The role of IRF in WSSV and poly (I:C)-stimulated induction of Vago4. (**E**) Interaction of IRF with the Vago4 promoter. EMSA was performed with purified recombinant IRF protein together with the wild-type and the ISRE-mutated sequences of the promoter region of Vago4. Super-shift assays were performed by adding anti-IRF or anti-6His tag antibodies into the EMSA system.

We analyzed the promoter regions of shrimp Vago genes^49^, and observed that an ISRE sequence can be predicted in the 5′-UTR of Vago4 but not in the other four Vago isoforms. To verify the transcriptional regulation effect of *L. vannamei* IRF on Vago promoters, dual luciferase reporter gene assays were performed on *Drosophila* S2 cells using luciferase-expressing vectors containing promoters of Vago isoforms (Fig. 4A). The results demonstrated that IRF could significantly up-regulate expression of Vago4 and Vago5 but not Vago1-3. We further detected the transcriptional regulation activity of IRF on promoters of Vago4 and Vago5 through improving the level of IRF expression in the dual luciferase reporter gene detection system (Fig. 4B). With the increase of the transfected IRF-expressing vector, the activities of the promoters of Vago4 and Vago5 were also enhanced, confirming that *L. vannamei* IRF could regulate the expression of Vago4 and Vago5.

It has also been reported that in insect Vago could be activated by viral nucleic acids^43^,^44^. We introduced poly (I:C) and WSSV treatments into the dual luciferase reporter gene detection system in order to investigate the role of *L. vannamei* IRF in activation of Vago by foreign nucleic acids. The Vago4 promoter was representatively detected. We observed that both poly (I:C) and WSSV could up-regulate the activity of Vago4 promoter (Fig. 4C,D). Without IRF expression, compared with the untreated control, the activity of Vago4 promoter was enhanced 6-fold after 6 h of WSSV treatment and 4-fold after 0.5 ug/mL poly (I:C) treatment. In the 30 ng IRF-expressing vector-transfected groups, the activity of Vago4 promoter was up-regulated 19-fold in the untreated cells, 40-fold in the WSSV treated cells, and 48-fold in the poly (I:C) treated cells. Obviously, compared with the IRF nonexpressing groups, overexpression of the *L. vannamei* IRF could enhance the effects of WSSV and poly (I:C) on the Vago4 promoter.

To further investigate the interaction between *L. vannamei* IRF and the Vago4 promoter, electrophoretic mobility shift assay (EMSA) was performed using purified 6His-tagged IRF protein expressed in *E. coli* cells together with wild-type or ISRE-mutated (as control) Vago4 promoters (Fig. 4E). IRF could retard the mobility of the wild-type Vago4 promoter DNA, but not the mutant one, and overloading of the mutant promoter DNA by 2- or 5- fold didn’t affect the DNA level in the protein/DNA complex of IRF/wild-type Vago4 promoter, suggesting that IRF could bind the IRSE sequence in Vago4 promoter. Moreover, the anti-IRF or anti-6his antibody was added into the EMSA system to bind the 6his-tagged IRF protein. We observed that these antibodies could further retard the mobility of the wild-type Vago4 promoter DNA to generate super shift bands, confirming the interaction between *L. vannamei* IRF and Vago4 promoter.

### Involvement of IRF in antiviral responses

**Figure 5:**
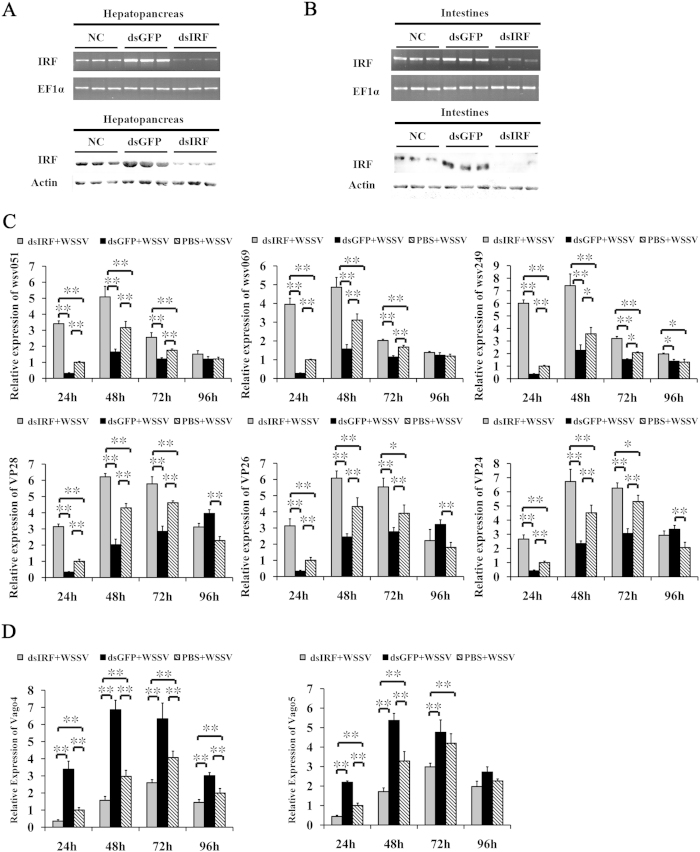
Knockdown of *L. vannamei* IRF *in vivo*. The RNA interference efficiencies in hepatopancreas (**A**) and intestines (**B**) were detected by both RT-PCR and western-blot. (**C**,**D**) Expression profiles of WSSV genes, wsv051, wsv069, wsv249, VP28, VP26 and VP24, and *L. vannamei* Vago4 and Vago5 in IRF-silenced shrimps during WSSV infection analyzed by real-time RT-PCR.

To investigate the role of *L. vannamei* IRF in the antiviral response, we knockdown the expression of IRF in living shrimps using RNAi strategy through injection of IRF-specific dsRNA. The knockdown efficiency was detected using RT-PCR and western-blot, which demonstrated that the IRF-specific dsRNA could significantly reduce the mRNA and protein levels of IRF in hepatopancreas and intestine (Fig. 5A,B). Interestingly, the control GFP dsRNA could obviously increase the expression of IRF, confirming that IRF could be activated by foreign nucleic acids.

After dsRNA or PBS treatment, shrimps were experimentally infected with WSSV. We detected the expression of WSSV functional genes, including immediate early genes wsv051, wsv069 and wsv249, and structural protein genes VP28, VP26 and VP24 using real-time RT-PCR (Fig. 5C). Expression of all these genes during 24–96 hpi was up-regulated in the IRF-dsRNA treated group but down-regulated in the GFP-dsRNA treated group, with the exception of the structural protein genes VP28, VP26 and VP24 in the GFP-dsRNA treated group at 96hpi, which demonstrated higher levels than those in the IRF-dsRNA treated group. Expression of the three immediate early genes wsv051, wsv069 and wsv249 in the IRF-dsRNA, GFP-dsRNA and the control PBS treated groups were all peaked at 48 hpi, while for the three structural protein genes, compared with the PBS control group, their expression peaks were advanced in the IRF-dsRNA group and postponed in the GFP-dsRNA group. These may suggest the antiviral effects derived by IRF on immediate early genes and structural protein genes of WSSV could be different, which needs further investigation. We further detected the expression of Vago4 and Vago5 after WSSV infection (Fig. 5D). In the PBS control group, expressions of Vago4 and Vago5 were up-regulated and both peaked at 72 hpi with 4.0- and 4.1-fold increase at 72 hpi compared with 0 hpi, respectively. In contrast, the expressions of Vago4 and Vago5 in the IRF-dsRNA group were significantly inhibited and peaked at 72 h with only 2.8- and 3.0-fold increase, respectively, while in the GFP-dsRNA group, they were significantly enhanced with expression peaks advanced to 48 hpi and reached 6.8- and 5.6-fold values, respectively. These confirmed that Vago4/5 expression could be induced by heterogeneous nucleic acids and be regulated by IRF.

**Figure 6:**
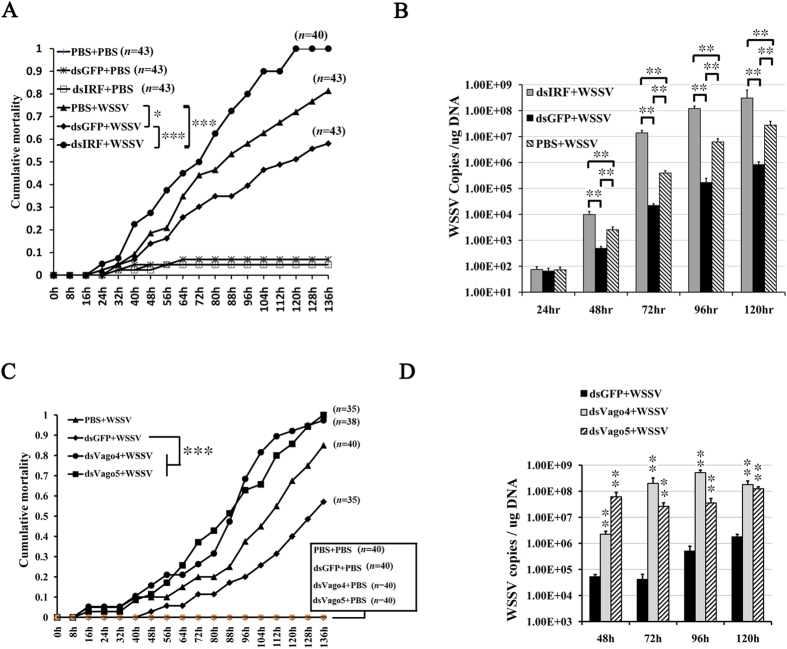
Function of IRF in shrimp antiviral immunity. Healthy *L. vannamei* were injected with IRF-, Vago4-, Vago5-, or control GFP-dsRNA and 48 h later were challenged with 10^6^ copies of WSSV particles or control PBS. The results were representative of three independent experiments, which gave nearly identical results. Mortalities of IRF- or Vago-silencing shrimps during WSSV infection were analyzed (**A**,**C**). Statistical significances between experimental and control groups were calculated using the Kaplan-Meier plot (log-rank χ^2^ test, *p < 0.05). WSSV genome copies in muscle tissue of IRF and Vago dsRNA treated shrimps at 24–120 h post infection (**B**,**D**). Statistical significances were calculated by the Student’s t-test (**p < 0.01). Bars indicate the mean ± SD.

A parallel experiment was also performed to record the mortality rates of IRF-knockdown shrimps after WSSV injection (Fig. 6A). Compared with the PBS control group, the cumulative mortality was significantly increased in the IRF-dsRNA treated group. Interestingly, we also observed that the mortality was decreased in the GFP-dsRNA treated group. Given that GFP-dsRNA could enhance the expression of IRF, this confirmed that IRF could be involved in the nucleic acid-induced antiviral immune responses of shrimp. We further investigated the virus load in shrimp muscle tissues using absolute quantative real-time PCR and observed that the WSSV DNA copies were significantly increased after IRF-dsRNA treatment and decreased after GFP-dsRNA treatment, consistent with the mortality result (Fig. 6B). In addition, to verify the role of Vago4/5 in antiviral responses, we knockdown the expression of Vago 4/5 in shrimps using RNAi strategy. The results demonstrated that suppression of Vago4/5 significantly increased the mortality of shrimps caused by WSSV infection and reduced the WSSV copies in the shrimp tissues, confirming that Vago plays important role in shrimp antiviral immunity (Fig. 6C,D).

## Discussion

Unlike IFNs, IRF-like genes have been widely predicted in invertebrate genomes^40^. A recent study has preliminarily showed that the pearl oyster IRF-2 (pfIRF) could activate promoters containing the IRSE or NF-κB binding site *in vitro*^41^. To our knowledge, it was the only report of the transcriptional regulatory function of an invertebrate IRF-like gene before the present study. However, the exact function of pfIRF in the immunity of pearl oyster has not been studied. The now available knowledge on invertebrate IRF genes, in particular their roles in antiviral immunity, is quite limited and needs further systemic investigation. In this study, the first crustacean IRF gene was identified in *L. vannamei*. The *L. vannamei* IRF protein contains a conserved IRF DNA-binding domain in the amino-terminus that is homologous to that of mammalian IRFs and serves as a DNA-binding motif, which has been confirmed by EMSA. IRF is mainly expressed in pyloric caecum, hepatopancreas and intestine but not in other tissues of shrimps, suggesting that it is not a ubiquitously expressed factor. Such specific tissue distributions suggest that signaling of the shrimp IRF pathway may occur in certain cells that were specifically present in these tissues. WSSV infection could lead to up-regulation and nuclear translocation of IRF, indicating that *L. vannamei* IRF could be activated in response to virus infection and thus is a virus-inducible transcriptional factor. Poly (I:C), a synthetic analog of viral dsRNA, could also activate the expression and translocation of IRF, suggesting IRF could be involved in the nucleic acid-inducible antiviral immune response of shrimp. Moreover, it has been reported that both mammalian IRF3 and IRF7 could form homodimmers, which are essential for their transcriptional regulatory functions^23^. In shrimp tissues, we also observed the dimerization of IRF protein, which was promoted upon WSSV or poly (I:C) stimulation. These suggest that *L. vannamei* IRF may possess the similar protein nature with mammalian IRFs.

The *L. vannamei* IRF could significantly activate the promoter containing a human ISRE sequence and ectopical expression of IRF in mammalian cells could specifically activate the promoters of human type I IFNs and successfully trigger an antiviral state in HEK293T cells against TFV infection. These exhibited that *L. vannamei* IRF could function in a similar way to mammalian IRFs in regulating immune responses, suggesting the transcriptional regulatory function of IRF could be conserved from invertebrates to mammals. Interestingly, in mammals, it has been known that type I and III IFNs share similar transcriptional regulatory mechanisms and the induction of type III IFNs is also regulated by IRFs^5^,^50^,^51^. For instance, expression of IL-29 gene is controlled by IRF-3 and IRF-7, resembling that of IFN-β, whereas IL-28 is mainly regulated by IRF-7, similar to that of IFN-α^51^. However, in this study, although *L. vannamei* IRF could significantly enhance the activity of IFN-α and -β promoters, it didn’t exert a regulatory activity on IL-29 and IL-28 promoters. This may suggest an additional preference of *L. vannamei* IRF with nuanced difference to that of mammalian IRFs, and further enlighten us that the regulatory mechanisms for the transcription of mammalian type I and III IFNs could differ in detail. These statements are worthy of further investigation.

Vago is a viral infection-inducible peptide firstly identified in *Drosophila* that can suppress the viral load of drosophila C virus in the fat body^44^. In *Culex* mosquito, Vago was found to be a secreted peptide that restricts West Nile virus (WNV) infection by activating JAK/STAT pathway, which is homologous to that in the mammalian IFN system^43^. Moreover, in insects, activation of Vago is dependent on sensing of viral dsRNA by Dicer-2, which belongs to the DExD/H-box helicase and is phylogenetically related to the mammalian RIG-I–like receptors^44^. It has been known that in mammalian cells RIG-I–like receptors functions to sense viral infection and mediate IFN induction. Thus, although Vago shared no sequence similarity with IFNs, it was considered to perform a similar function to Type I IFNs^52^. In this study, the *L. vannamei* Vago4 and Vago5 genes were found to be controlled by IRF in a similar manner to that of IFNs. Therefore, our finding supports the opinion that Vago could function as an IFN-like molecule in invertebrates^43^. The other Vago isoforms could not be controlled by IRF and their regulatory mechanisms need further investigation, suggesting that the *L. vannamei* Vago genes could have undergone differentiation in regulatory mechanisms. It has been reported that in *Culex* mosquito, Dicer-2-dependent activation of Vago could be controlled by the TRAF-Rel2 (a NF-κB ortholog) pathway^45^. The regulatory effect of NF-κB on *L. vannamei* Vago genes also needs further investigation. Moreover, in insects, it has been known that Vago could not be activated by poly (I:C), whereas in this study we observed that ploy (I:C) could efficiently promote the expression and dimerization of *L. vannamei* IRF *in vivo*. As the IRF homology sequence has not been found in insect genomes and only one Vago gene has been identified in *Drosophila* or *Culex* mosquito up to now, the difference of the regulatory mechanism between insect and crustacean Vago genes is worthy of in-depth studies. In addition, whether Dicer-2 also functions as a receptor to implicate in the activation of shrimp Vago requires further investigation.

**Figure 7:**
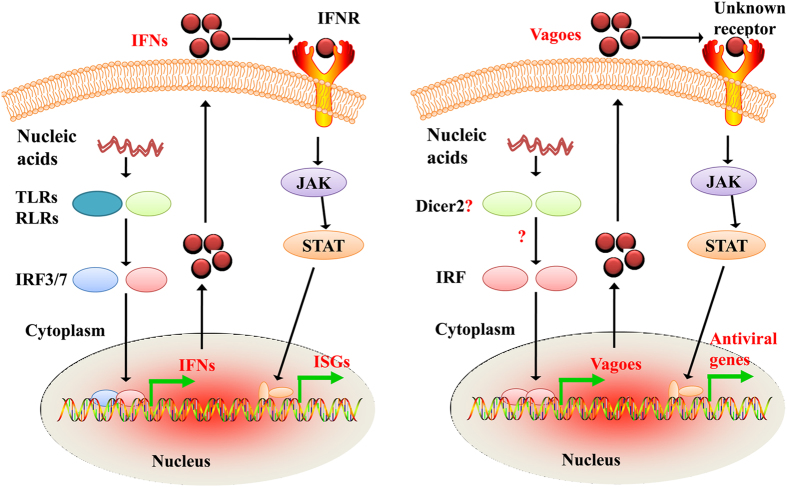
Diagram of activation of the vertebrate IFN system (left panel) and the crustacean Vago system (right panel). (C.L. drew the figure).

Appearance of the IFN response is a hallmark of the evolution of antiviral immunity. At present, there is a debate on whether invertebrates possess an antiviral immunity similar to the IFN system of vertebrates. It is clear that in vertebrates, sensing of pathogen invasion, transduction of signals, induction of IFN expression, engagement of IFN receptors, activation of the JAK/STAT pathway, expression of ISGs and establishment of the antiviral cellular state as well as the associated feedback regulatory mechanisms constitute the process of the establishment of IFN-mediated innate immune responses, orchestrated by multiple cellular receptors, signal transductors, regulators and effectors, and centered on the IRF-IFN-JAK/STAT axis (Fig. 7, left panel)^5^,^6^. Interestingly, it has been reported that shrimps, in particular *L. vannamei*, possess nucleic acid-induced antiviral immunity, and a number of critical components of the shrimp antiviral response have been identified, including IKK-ε, Dicer-2, Toll-like receptors, and many components of the JAK/STAT pathway^33^,^53^,^54^. We also demonstrated here that *L. vannamei* Vago genes are essential for shrimp antiviral immunity. Therefore, based on these, an elementary outline of the Vago system that plays a critical role in nucleic acid-induced antiviral immunity can be sketched in shrimps, which is also centered on the IRF-Vago-JAK/STAT axis (Fig. 7, right panel). As Vago has been suggested to be an IFN-like protein and IRFs to be conserved from invertebrates to mammals, it could be seen that the shrimp Vago system exhibits similarity to the mammalian IFN system, indicating a possible evolutionary relation between them. In summary, our study hints that invertebrates, especially shrimps, might possess an IFN system-like antiviral response, which is interesting and worthy of in-depth studies.

## Methods

### Animal and pathogens

The animal use protocol listed below has been reviewed and approved by the Animal Ethical and Welfare Committee (AEWC) of School of Life Sciences, Sun Yat-sen University. All animal experimental procedures were performed in accordance with the Regulations for the Administration of Affairs Concerning Experimental Animals approved by the State Council of People’s Republic of China. Healthy *L. vannamei* (average 5 g) were collected from Hengxing shrimp farm in Zhanjiang city, China, cultured in a recirculating water tank system filled with air-pumped sea water with 2.5% salinity at 27 °C, and fed with a commercial diet at 5% of body weight per day. WSSV is prepared from the muscle tissue of WSSV-infected shrimps and stored at −80 °C. Muscle tissue was homogenized and prepared as WSSV inoculum to a final concentration (1 × 10^5^ virions/50 μL) following a published protocol^55^.

### Cloning of IRF cDNA

Shrimp total RNA was extracted using RNeasy Mine Kit (Qiagen, Germany) and reverse transcribed into cDNA using a PrimeScript^TM^ RT reagent kit (TaKaRa, Japan). A partial cDNA sequence homologous to mammalian IRFs was retrieved from the sequenced *L. vanname* transcriptome data^56^, and primers IRF-5RACE1 and IRF-3RACE1 were then designed to receive the 3′ and 5′ ends of *IRF* cDNA sequences by rapid amplification of cDNA ends (RACE). PCR program was set as described before^57^. The PCR products were used as templates with the primers IRF-5RACE2 and IRF-3RACE2 for the secondary PCR, with the PCR conditions the same as above. The PCR products were then cloned into the pMD-19T vector (TaKaRa, Japan) and sequenced. The sequences were analyzed and deposited in the NCBI GenBank (GenBank accession no. KM277954).

### Vectors

The open reading frame (ORF) of *L. vannamei* IRF without termination codon was cloned in frame into pCDNA3.1, pAc5.1 and pAc5.1-GFP vectors to generate pcDNA-IRF, pAc-IRF-V5 and pAc-IRF-GFP, respectively. The promoter regions of human type I (IFN-α, –β, –ω) and type III IFNs (IL28 and IL29) as well as five *L. vannamei* Vago isoforms (Vago1 ~ 5)^49^ were retrieved from Genbank (Supplementary Data1) and cloned into the PGL-3 vector (Promega, USA) to generate luciferase reporter gene vectors.

### Antibodies

The *L. vannamei* IRF ORF was cloned in to pET-32a (+) plasmid (Merck Millipore, Germany). Recombinant plasmid was transformed into BL21(DE3) *Escherichia coli* strain to express Trx-IRF fusion protein. Recombinant protein was purified with Ni-NTA agarose (Qiagen, Germany) and separated by electrophoresis in 12% SDS-polyacrylamide gels. The gel slice containing Trx-IRF band was cut out and ground with adjuvant to immunize New Zealand rabbits to produce anti-IRF antibodies. Anti-Mx and -Tubulin antibodies were purchased from Abcam (UK), and the anti-β-actin antibody from Merck Millipore (Germany).

### RT-PCR, native-PAGE and western-blot

For tissue distribution analysis, hepatopancreas, pyloric caecum, hemocyte, gill, stomach, intestine, epidermis, and muscle from 15 healthy *L. vannamei* were sampled, pooled and subjected to RNA purification and cDNA reverse-transcription as previously described^57^. RT-PCR and real-time PCR was then performed for quantification of IRF expression with EF-1α gene detected as internal control.

To analyze the expression profile of IRF after challenges, *L. vannamei* were divided into 3 groups (n = 50), in which shrimps were injected at the second abdominal segment with PBS (as control), 5 μg Poly (I:C) or 1 × 10^5^ copies newly extracted WSSV particles. Hepatopancreas of challenged shrimps were sampled at 0, 4, 8, 12, 24, 36, 48, 72 h post injection (hpi) with each sample collected and pooled from 15 shrimps. Samples were subjected to RNA extraction and real-time PCR analysis for detection of IRF expression. For dimmer protein analysis, samples were analyzed using native-PAGE and further translocated into NC membrane and detected by western-blot.

### Co-immunoprecipitation and Western-blot assays

The pAc-IRF-V5 vector was co-transfected with pAc-IRF-GFP or pAc5.1-GFP (as control) into *Drosophila* Schneider 2 (S2) cells. After 72 h, cells were harvested and lysed in NP-40 lysis buffer with a protease inhibitor cocktail (Sigma). Co-immunoprecipitation and reciprocal co-immunoprecipitation were performed using anti-V5 affinity gel (Sigma) and anti-GFP agarose (MBL International), respectively. Western-blot was then performed with rabbit anti-GFP antibody or mouse anti-V5 antibody (Sigma) as primary antibody, and alkaline phosphatase-conjugated goat anti-rabbit as secondary antibody (Sigma).

### Confocal laser scanning microscopy

To detect the subcellular localization of IRF, S2 cells were transfected with GFP-fused IRF using Effectene Transfection Reagent (Qiagen, Germany). At 24 h post-transfection, cells were treated with 10^5^ copies/mL of WSSV, 1 μg/1mL of poly (I:C), or PBS (as control), and 6 h later, cells were stained with 2 μg/ml Hochest 33258 (Sigma, USA) and visualized on a confocal laser scanning microscope (Zeiss Axio Cam Mrm; LePecq, France) and analyzed using Axio-Vision software version 4.6 (Carl Zeiss).

### Dual-luciferase reporter assays

To detect the effects of *L. vannamei* IRF on promoters of human IFNs and *L. vannamei* Vago genes, dual-luciferase reporter assays were performed on HEK293T cells and S2 cells using pcDNA-IRF and pAc-IRF-V5 as IRF-expressing vectors, respectively. Briefly, cells in each well of a 96-well plate (TPP, Switzerland) were transfected with 0.05 μg reporter gene plasmids, 0.005 μg pRL-TK renilla luciferase plasmid (Promega), and 0.05 μg expression plasmids or empty expression vectors (as control). The pRL-TK renilla luciferase plasmid was used here as an internal control. At 48 hour post transfection, Dual-Luciferase Reporter Assays were performed to measure the firefly and renilla luciferase activities according to the manufacturer’s instructions with each experiment done in triplicates.

### Detection of antiviral effects

The HEK293T cells were transfected with pcDNA-IRF and pcDNA3.1 (as control) and 24 h later were infected with TFV at MOI = 3. Pathological signs of cells at 24, 48 and 72 h post infection were investigated using microscopy. Titers of the released TFV in the cell supernatants was analyzed using the TCID50 method as previously described^58^.

### Double-stranded RNA-mediated RNA interference

The dsRNAs targeting the full lengths of IRF, Vago4, Vago5 and GFP (as control) genes were synthesized by *in vitro* transcription according to the protocols as previously described^59^. To investigate the RNA interference efficiency, healthy *L. vannamei* were injected at the second abdominal segment with 10 μg IRF, Vago4/5 or GFP dsRNA (in 50 μl PBS) or PBS (as control), and sampled for hepatopancreas tissues at 72 h post-injection with 9 shrimps in each sample. The mRNA level of IRF was detected by RT-PCR three times (each with RNA pooled from 3 random shrimps in a sample) using primer pairs QIRF-F/QIRF-R (Table S1) with the elongation factor 1 alpha (EF1-α) gene amplified as internal control using LvEF-1α-F/LvEF-1α-R (Table S1). The protein level of IRF was examined by western-blot as described above with β-actin as control.

For pathogen challenge tests, healthy *L. vannamei* were injected with dsRNA and 48 h later were challenged with 10^5^ copies WSSV particles in 50 μL PBS, and mock-challenged with PBS as a control. Shrimps were kept in culture flasks for 7 days following infection. Experiments were repeated in triplicate and the cumulative mortality was recorded every 8 h and subjected to statistical analysis using MedCalc statistical software version 11.2 (Mariakerke, Belgium) to generate the Kaplan-Meier plot (log-rank χ^2^ test).

To analyze the viral load and viral gene expression in tissues, the parallel WSSV challenge experiments were also performed and muscle and intestine tissues were sampled from surviving shrimps at 24, 48, 72, 96 and 120 h post infection (hpi) with 10 shrimps in each sample. WSSV copies in muscle tissues were assessed by absolute real-time quantitative PCR using primers vp28-qF/vp28-qR as previously described^55^. Transcription levels of shrimp Vago4/5 genes and multiple WSSV genes were analyzed using real-time PCR with specific primers (Table S1).

### EMSA

EMSA was performed according to the method of Huang *et al.*^60^. Briefly, the wild-type and the ISRE-mutated sequences of the 5′-UTR of Vago4 were purified by gel electrophoresis and dissolved in PBS buffer. And 1 μg of the products were each mixed with 50 μL PBS containing 1 μg purified recombinant IRF protein and incubated at 27 °C for 1 h. For super-shift assays, anti-IRF or -6His tag antibodies were further added into the system before incubation. The mixtures and controls (without DNA or protein in the system) were then analyzed using gel electrophoresis.

## Additional Information

**How to cite this article**: Li, C. *et al.* Activation of Vago by interferon regulatory factor (IRF) suggests an interferon system-like antiviral mechanism in shrimp. *Sci. Rep.* **5**, 15078; doi: 10.1038/srep15078 (2015).

## Supplementary Material

Supplementary Information
