# Supplementary material for: Activation of Vago by interferon regulatory factor (IRF) suggests an interferon system-like antiviral mechanism in shrimp
Source: Sci Rep. 2015 Oct 13;5:15078. doi: 10.1038/srep15078 (PMC4602278; doi:10.1038/srep15078)
Supplement: Supplementary Information [file srep15078-s1.pdf]

Activation of Vago by interferon regulatory factor (IRF) suggests an interferon system-like antiviral mechanism in shrimp

Chaozheng Li [1, 2, 4], Haoyang Li [1, 2, 4], Yixiao Chen [1, 2, 4], Yonggui Chen[1, 3, 4], Sheng Wang [1, 2, 4], Shao-Ping Weng [1, 2, 4], Xiaopeng Xu [1, 2, 4, *], Jianguo He [1, 2, 3, 4, *]

1. MOE Key Laboratory of Aquatic Product Safety / State Key Laboratory for Biocontrol, School of Life Sciences, Sun Yat-sen University, Guangzhou, P. R. China

2. Institute of Aquatic Economic Animals and Guangdong Provice Key Laboratory for Aquatic Economic Animals, Sun Yat-sen University, Guangzhou, P. R. China

3. School of Marine Sciences, Sun Yat-sen University, Guangzhou, P. R. China

4. South China Sea Resource Exploitation and Protection Collaborative Innovation Center (SCS-REPIC), Guangzhou, P. R. China

A

```
   1 atcgggatccactcgcagatacagatctctctctctctctctctctctctacctgccttcctcgctgtctctctctctcttattcgca
  91 ctttgtgtttctgtaattcgcaccaggtatttgtttgcttcattggagttgtttcggaatctcggatctcgctgcttcactgcttcggat
   1                M  P  P  S  F  T  N  V  P  M  D  L  S  M  K  S  R  R  K  L  R  L  E
 181 aactcgtcggtctaaggtcgccATGCCGCCATCTTTCACCAATGTCCCGATGGATCTGTCCATGAAATCGCGCCGAAAGCTCCGTCTGGA
  24 E  F  L  R  Q  N  L  D  E  A  P  A  G  R  V  I  E  W  V  D  R  A  K  G  I  F  R  I  L  W
 271 GGAGTTCCTGCGCCAGAACCTGGACGAGGCTCCTGCAGGGCGCGTGATCGAGTGGGTGGACCGCGCCAAGGGCATCTTCAGGATTCTGTG
  54 T  H  Q  S  S  G  A  F  T  H  D  D  A  A  L  F  R  Y  W  A  L  A  R  G  K  P  S  N  L  S
 361 GACGCACCAGAGCTCGGGGGCATTCACGCATGACGACGCTGCCCTCTTTCGCTACTGGGCTCTTGCTCGGGGAAAACCATCCAACCTGTC
  84 S  V  E  L  K  Q  S  L  R  M  A  L  N  K  S  P  S  V  E  R  I  E  M  T  H  D  E  Y  R  Y
 451 TTCAGTGGAGCTAAAGCAGAGTCTGCGAATGGCCCTGAATAAGTCGCCTTCAGTAGAACGCATAGAGATGACACATGACGAATATCGATA
 114 F  R  F  T  A  W  S  S  A  K  S  S  S  R  P  V  P  V  A  S  S  H  S  Y  D  I  A  A  D  F
 541 CTTCAGGTTCACAGCGTGGTCCTCGGCCAAGTCCTCCTCCCGCCCCGTCCCCGTCGCCTCCTCGCATTCCTACGACATCGCCGCCGACTT
 144 A  S  S  S  P  K  V  R  K  V  Q  G  P  E  D  A  L  P  P  T  R  E  H  R  P  L  L  R  D  L
 631 CGCCTCCTCCAGCCCCAAGGTCCGGAAGGTCCAGGGCCCCGAGGACGCCCTCCCGCCCACGAGGGAACACCGGCCTCTCCTCAGGGACCT
 174 L  T  Q  P  D  T  T  A  H  D  P  Y  S  S  K  R  V  V  S  P  P  A  S  T  S  S  S  E  D  A
 721 TCTAACCCAGCCCGACACGACCGCCCACGACCCCTACTCCTCCAAGAGGGTCGTCAGCCCCCCTGCCAGCACCTCGTCGAGTGAGGACGC
 204 L  S  W  C  S  G  P  E  D  T  A  P  R  Y  A  F  E  P  P  T  T  C  P  V  R  D  D  P  S  P
 811 GCTGTCGTGGTGTTCCGGGCCCGAGGACACGGCTCCGCGGTACGCCTTCGAACCCCCCACGACCTGTCCCGTGAGAGACGACCCCAGCCC
 234 P  E  M  D  D  Y  F  G  R  R  V  P  T  K  D  D  L  P  P  R  S  I  A  L  R  T  D  L  Y  D
 901 GCCCGAGATGGACGACTACTTCGGCCGCCGCGTCCCCACGAAGGACGACCTCCCGCCAAGGAGCATCGCCCTGCGCACCGACCTGTACGA
 264 P  T  A  K  T  D  P  Y  H  L  Y  L  H  G  H  S  Y  D  L  A  E  K  P  K  H  Y  D  P  W  T
 991 CCCGACCGCCAAGACCGACCCCTACCACCTCTACCTGCACGGCCACAGCTACGACCTCGCCGAGAAACCGAAGCACTACGACCCCTGGAC
 294 A  C  S  P  R  E  N  S  E  V  Q  C  A  V  P  S  L  S  D  C  L  Q  P  G  F  D  H  Y  P  S
1081 CGCTTGCTCCCCGCGCGAGAACAGTGAGGTGCAGTGCGCGGTCCCGTCCCTCAGCGACTGCCTCCAACCGGGTTTCGATCACTACCCGAG
 324 F  Y  P  S  S  F  P  S  V  S  S  S  F  R  L  D  G  S  P  L  F  N  S  Y  F  M  R  G  R  H
1171 CTTCTATCCGAGTAGTTTCCCTTCGGTCTCCTCGAGCTTTCGTCTGGATGGCTCGCCGCTCTTTAACAGCTATTTTATGAGGGGGAGGCA
 354 L  Y  A  G  E  R  T  L  P  *
1261 CTTGTATGCCGGCGAGAGGACGTTGCCGTAGacgaactcaaattaatcatcatttttgtatataacgatagctttttttttgcggtttaaa
1351 tatttgtataattatatgtaaatatatctatatctacagcaaaaaaaaaaaaaaaaaaaaaaaaaa
```

B

```
                 *       20        *       40        *       60
LvIRF  : KSRRKLRLEEFLRQNLDEAPAGRVIEWVDRAKGIFRILWTHQS-SGAFTHDDAALFRYWALARG :  63
PoIRF  : MPVSRMRMRPWLEKMIESNT-ISGLTWVDKDQKMFSIPWKHAARHGWELDKDASLFKKWAIHTG :  63
TrIRF  : MPVSRMRMRPWLERMINSDT-IEGLKWVDQAKTMFSIPWKHAARHGWEIEKDANLFRMWAIHTG :  63
AcIRF1 : MPQARLRMRPWLEKQINSNR-FDGLVWLNKEQKLFQIPWKHAAKHGWDLEKDASLFKSWAIHTG :  63
DrIRF1 : MPVSRMRMRPWLESRIDSNT-INGLMWVNKEEKMFSIPWKHAARHGWEVDKDACLFKQWAIHTG :  63
EcIRF1 : MPVSRMRMRPWLEQQIESKA-VSGLNWVDKEKTMFSIPWKHAARHGWELDKDASLFKQWAIHTG :  63
HsIRF1 : MPITRMRMRPWLEMQINSNQ-IPGLIWINKEEMIFQIPWKHAAKHGWDINKDACLFRSWAIHTG :  63
XlIRF1 : MPVTRLRMRPWLEEQINSNK-IPGLSWINKDKMIFQIPWKHAARHGWDINKDACLFRSWAVHTG :  63
CgIRF2 : RPIERQKMRPWLVNLLNNEK-VHGFSWVSKDHETFRISWRHAARQGWDPIMDAGLFERWAKHTG :  63
GbIRF2 : MPVERMRMRPWLEEQINSNK-IPGLKWLNEEKKIFQIPWMHAARHGWDVEKDAPLFRNWAIHTG :  63
GgIRF2 : MPVERMRMRPWLEEQINSNT-IPGLKWINKEKKIFQIPWMHAARHGWDVEKDAPLFRNWAIHTG :  63
HcIRF2 : RPLERQRMRPWLIELLDKDT-MSQLSWLNKREKMFRITWRHAARQGWDMAKDACLFEKWARHTG :  63
HsIRF2 : MPVERMRMRPWLEEQINSNT-IPGLKWLNKEKKIFQIPWMHAARHGWDVEKDAPLFRNWAIHTG :  63
PfIRF2 : RPIERQKMRSWLMELLKSDRIIAGFGWFSKKEKSFRISWRHAARHGWDPETDASLFELWAKHTG :  64
XlIRF2 : MPVERMRMRPWLEEQINKDL-IPGLKWLNKEKKIFQIPWMHAARHGWDVDKDAPLFKNWAVHTG :  63
          p  4 46rp5L   6         g  W          F I W Haa hGw   kDA LF   WA htG

                 *       80        *       100        *                  Identity  Similarity
LvIRF  : KP----SNLSSVELKQSLRMALNKSPSVER-----IEMTHDEYRYFRFTAW : 105     —          —
PoIRF  : KYTEGQTS-DPKTWKANFRCAMNSLPDIEEVKDKSIHKGQQAVRVFKMLHV : 113    23%        43%
TrIRF  : KYVEGQAN-DPKTWKANFRCAMNSLPDITEVKTMSVNKGHQAVRVFKMLPA : 113    26%        43%
AcIRF1 : RYKEGDVQPDPKTWKANFRCAMNSLPDIEEVKDKSISKGSSAVRVYRMLET : 114    21%        42%
DrIRF1 : KYKEGVTQPDPKTWKANFRCAMNSLPDIEEVKDKSINKGCGAVRVYRMLPA : 114    23%        40%
EcIRF1 : KYVEGQAC-DPKTWKANFRCAMNSLPDIEEVKDRSINKGHQAVRVFRMLPA : 113    26%        42%
HsIRF1 : RYKAGEKEPDPKTWKANFRCAMNSLPDIEEVKDQSRNKGSSAVRVYRMLPP : 114    21%        40%
XlIRF1 : RFKSGEKETDPKTWKANFRCAMNSLPDIKEVKDKSIYKGSSAVRVYQMFTP : 114    22%        41%
CgIRF2 : KYVDGDEP-DPKRWKANFRCALNSLPDVKQLKDQGQRKGKDAYKVYQFLNE : 113    24%        37%
GbIRF2 : KYQPGVDKADPKTWKANFRCAMNSLPDIEEVKDKSIKKGNNAFRVYRMLPL : 114    24%        44%
GgIRF2 : KYQSGVDKPDPKTWKANFRCAMNSLPDIEEVKDKSIKKGNNAFRVYRMLPL : 114    24%        45%
HcIRF2 : KFYDGDKP-DAKRWKANFRCALNSLPDVVEEKSKGMKKGQNAFKVYRFLDD : 113    24%        44%
HsIRF2 : KHQPGVDKPDPKTWKANFRCAMNSLPDIEEVKDKSIKKGNNAFRVYRMLPL : 114    24%        45%
PfIRF2 : KFIDGDEP-DPKRWKANFRCALNSLPDVVEVRESSVRKGVNAYRVYKFLDE : 114    23%        37%
XlIRF2 : KYQPGIDKPDPKTWKANFRCAMNSLPDIEEVKDKSMKKGNNAFRVYRMIPV : 114    22%        46%
         4   g    dpk wKanfRcA6NslPd6       s  kg  a 4v5
```

Figure S1. Sequence analysis of *L. vannamei* IRF. (A) Nucleotide and deduced amino acid sequences of *L. vannamei* IRF. The DNA-binding domain was shaded. (B) Multiple sequence alignments of the DNA-binding domains of IRFs. The identical amino acid residues were shaded in black and the similar residues in gray. Proteins analyzed list below: LvIRF, *Litopenaeus vannamei* IRF (Assession No. KM277954); PoIRF, *Paralichthys olivaceus* IRF (Assession No. BAA83468.1); TrIRF, *Takifugu rubripes* IRF (Assession No. AAK28340.1); AcIRF1, *Anolis carolinensis* IRF1 (Assession No. XP_003217436.1); DrIRF1, *Danio rerio* IRF1 (Assession No. NP_991310.1); EcIRF1, *Epinephelus coioides* IRF1 (Assession No. ACF95885.1); HsIRF1, *Homo sapiens* IRF1 (Assession No. NP_002189.1); XlIRF1, *Xenopus laevis* IRF1 (Assession No. NP_001083250.1); CgIRF2, *Crassostrea gigas* IRF2 (Assession No. EKC43156.1); GbIRF2, *Gloydius brevicaudus* IRF2 (Assession No. ADL09139.1); GgIRF2, *Gallus gallus* IRF2 (Assession No. NP_990527.1); HcIRF2, *Hyriopsis cumingii* IRF2 (Assession No. AEG21070.1); HsIRF2, *Homo sapiens* IRF2 (Assession No. AAP35928.1); PfIRF2, *Pinctada fucata* IRF2 (Assession No. JX863899.1); XlIRF2, *Xenopus laevis* IRF2 (Assession No. NP_001088726.1).

Table S1. Summary of Primers in this study.

**Name**	**Sequence (5’–3’)**
**RACE**	
LvIRF-3RACE1	GAGGACGCGCTGTCGTGGTGTTCC
LvIRF-3RACE2	ACCTCTACCTGCACGGCCACAGCTA
LvIRF-5RACE1	TGCGTTCTACTGAAGGCGACTTATTCAG
LvIRF-5RACE2	GCTCCACTGAAGACAGGTTGGATGG
**RT-PCR**	
LvIRF-F	CTTTCGCTACTGGGCTCTTGC
LvIRF-R	GGTCGTAGTGCTTCGGTTTCTC
LvEF-1α-F	TCGCTTCAAGGAAATCCACAAG
LvEF-1α-R	AAGGTCTCCACGCACATAGGC
LvActin-F	ACGAAGTAGCCGCCCTGGTTG
LvActin-R	GGTGGTCGTGAAGGTGTAGCC
**Real-time RT-PCR**	
LvIRF-F	ACGCTGCCCTCTTTCGCTAC
LvIRF-R	ACGCTGTGAACCTGAAGTATCG
LvEF-1α-F	GTATTGGAACAGTGCCCGTG
LvEF-1α-R	ACCAGGGACAGCCTCAGTAAGA
**Protein expression (pAc5.1A and pCDNA3.1A)**	
LvIRF-F	GG*GGTACC*ATGCCGCCATCTTTCACCAATG
LvIRF-R	GG*TCTAGA*CTACGGCAACGTCCTCTCGCCGGCA
**Protein expression (pET-32a (+))**	
LvIRF-F	CGGGATCCATGCCGCCATCTTTCACC
LvIRF-R	CCCAAGCTTACTACGGCAACGTCCTCTCG
**dsRNA templates amplification**	
dsRNA-LvIRF-T7-F	GGATCCTAATACGACTCACTATAGGATGCCGCCATCTTTCACCAATG
dsRNA-LvIRF-R	CTACGGCAACGTCCTCTCGCCGGCA
dsRNA-LvIRF-F	ATGCCGCCATCTTTCACCAATG
dsRNA-LvIRF-T7-R	GGATCCTAATACGACTCACTATAGGCTACGGCAACGTCCTCTCGCCGGCA
dsRNA-GFP-T7-F	GGATCCTAATACGACTCACTATAGGATGGTGAGCAAGGGCGAGGAGCT
dsRNA-GFP-R	TTACTTGTACAGCTCGTCCATGC
dsRNA-GFP-F	ATGGTGAGCAAGGGCGAGGAGCT
dsRNA-GFP-T7-R	GGATCCTAATACGACTCACTATAGGTTACTTGTACAGCTCGTCCATGC

Data file S1. Sequences of related gene mRNAs, promoter regions and primers for pGL3 luciferase reporter vector cloning. The transcriptional starting sites are marked with red letters.

>*Litopenaeus vannamei* Vago1 mRNA (Accession No. HQ541158.1)
CGGCCGGGGCACATCAAGTATTTTCAGTTGGTGTTCATATTTGCCACAGCCATGAA
GTTCTTGCTGATTGCTTGCTTGGGAAGCCTCGTCTTCGCACAACAGGGACCGGCT
GACCTGCAGGGCCCAGGGCCCTTCGTCAGGGACCTGAAAGCTGACGTGCTGAGA
GACTTCCCAGAACTTTGCTTCTCGTCAACAAACTTCCGACTCTTCCTGGAAAATCA
GTCCTGGAGCTTGTTCCCCTTCTGCGGCAAAGCTGAATGCGTCAAGAGCGGCGCA
GACTACATTGAGCGCGTCCATGACTGCGGTCCTCAGCCCAAGAATGCTGAAGCAT
GTACAATTGCCAACCTAGCTGAGCTTCAACGGAACGACACCATCCTCGAATACCCA
TCGTGCTGCCCCAAATACGTGTGCCCTGATGGAGTCACCCTTGAATATCCTACCCA
GGAAGAGATCAAGGCTGAGATTCAGAAGCAAAACCAAGCCGCTCTGCAGGCAGC
CAAGGAGGCCGCAGCAGCAAGGGAAGCGGCAGGCCCACAGGCAGCACCAGGAA
CTGCTTAAGTTACTCCAGAAAGACGAGGCAAAATAAACTGTAATGATAATAAAGTT
ATTTGGATAATAAAAAAAAAAAAAAAAAAAAAAAAAAAA

>Promoter of *L. vannamei* Vago1
GCACGCACTAAAAGCACTCACGTATGCACGTCCACACACAAACTCACATACATATA
TAGATACATATACACATACATACATACATATATGATTTTATATGTTATCATTTTTGGATT
CAGTAAAACCGTTTCTACAATATTAATTTCTTTTGTGATTTGGAAATTAAGTTTCTTC
AGAGGCGATCGGACTGCCACAGTGGGAATGCCACCAGATATCGAATTTGGGCTGA
GATTTAAAATAAAACAATTCAACAATGATTTTCACATACAAATGCAGCTCTAACAA
ATTATTTTACTAGGAAGTATATTTAGTGCGAAGTTATTCATCAAATGGATAAGTGCAT
AACGAATGACAAAGTAAAACAGACTACCAATATAAATTTCTAAATGGAATCTCTAT
CACGCAGGCGCAGGGAGAACGTCCATGAGTTTCGTGCATATAAAAGCCGCGGTTT
CATCGACGAGCTTCGGCCGGGGCACATCAAGTATTTTCAGTTGGTGTTCATATTTG
CCACAGCCATGAAGTTCTTGCTGATTGCTTGCTTGGGAAG

Primers:
pGL3-LvVago1-F:TTA*GAGCTC*GCACGCACTAAAAGCACTCA
pGL3-LvVago1-R:GTT*CTCGAG*CTTCCCAAGCAAGCAATCAG

>*Litopenaeus vannamei* Vago2 mRNA (Accession No. HQ541159.1)
ACAGCCACCACGATAAGCTTACAGTTCATAGAAAATACATGAGTTAACTAGAAATA
GCTGACACAGGCCGGTCCACTCTAGAGAAAAGGCCCGGGCGAAGCTAGTCGTCG
CAAATCTAACTGAACTGAACTGAAGTGAGCCACAGATTCCCAGGTCTCAGCAGCC
CCGTCCTCAGGCAGCACCAGCAGCTAGTCACTATGGCGTCTCTTTTGAGGATCCTG
TCCCTCCTTGCTGTTGTCGGAGCGTCCTTCGCAGCGGCTTACATTGGCCCAGCAGA
GGTTCACAAAGACTTCCCTGGCAAGTGCTATATCGCAAGCATAGGAGCCGTCCTTC

CCCTGGGCTACTCGTTGCAGGTGCGCAGCGTGTGCGAGACGAAGACCTGCATTGA
ACAAAGCGGAGATCTTTATCTGGAGGAAATAGGCTGCGGCTTGGTGGTCCAGTAC
GAAAACTGCAGAGTCGTGGAGGACAAATCAAAGCCTCACCCCTACTGCTGCCCC
AACTTGGAATGTAACTAAACGAAGACGAAGGTTTAAACAAAGGGAAATGTTTATC
GCAGTTTTTTTCTCTCCTTTTCTTTGTTTCGTTTGTAATTCTATTTCTTTTCAGTGG
GGTAAGAATAAAAAAGATTGATCAAGATGGGCCTTTTAATGTTTCGTGGAATTTTG
TGTGTGTGTTTTCTTGACTCTTAACATTTTTTCTGATTTGTTAAATATAGTTAGAGCT
TCCCTATAAAAAAAAAAAAAAAAAAAAAAAAAAA

>Promoter of *L. vannamei* Vago2
ACTATAGGGCACGCGTGGTCGACGGCCCGGGCTGGTCTGGCAGGTCAGCTAGAGG
AGAGGTTGCAACCATGGGAATCCCAGCCAGGTAGCCTAAGGCAGAGCGTTCTGTG
GGGACTCTTAAAACATGAATGCAGAGTCTGGTTTGTGTGTGTGTGTGTGTGTGT
GTGTGTGTGAATATAGATATAGATATACACATACATTGTACACACATTGTACAAGCA
TCTGGGCAAAGAAAGAAACAAAGAAGAGCGAAGGGGCGGGAGGAAGGGAGGG
GCTTAAGCGGCGGCAGGTATATAAAGGGGTCCTCGCTTGCTTGGACTACAGCCAC
CACGATAAGCTTACAGTTCATAGAAAATACATGAGTTAACTAGAAATAGCTGACAC
AGGCCGGTCCACTCTAGAGAAAAGGCCCGGGCGAAGCTAGTCGTCGCAAATCTA
ACTGAACTGAACTGAAGTGAGCCACAGATTCCCAGGTCTCAGCAGCCCCGTCCTC
AGGCAGCACCAGCAGCTAGTCACTATGGCGTCTCTTTTGAGGATCCTGTCCCTCCT
TGCTGTTGTCGGAGCGTCCTTCGCAGCGGCTTACATTGGCCCAGCAGAGGTTCAC
AAAGACTTCCCTGGCAA

Primers:
pGL3-LvVago2-F:AAT*GGTACC*ACTATAGGGCACGCGTGGT
pGL3-LvVago2-R:TTC*AGATCT*TTGCCAGGGAAGTCTTTGTG

>*Litopenaeus vannamei* Vago3 mRNA (Accession No. HQ541160.1)
AGGTCTCAGCAGCCCCGTCCTTAAGCACCACCAGCAACAAGTCACTATGGCGTCT
CTTTTGAGGGTCTTGTTCTTCCTTGCTGTTGTCGGAGTGTCTTTCGCTGCGCTTTTC
AGAGGGAAAGCAGAGGTTCACAAAGACTTCCCTGGTAAGTGCTATGTCGCAAGC
CTAGGAACCGGCTTTACCCCGGGTTCCTCGTGGCCCATGAGTGAGAAGTGCGCGA
AGAGGACCTGTTATGAAGAAGACGGAGAACTTTATTTCGAGGAAGCAAGCTGCG
GCGTGGCGATCCCGTCCGCAAACTGCAGAGTTGTGGAAGAAACAACGCTGCCTC
ACCCCTACTGCTGCCCCAGGCTGGACTGTAACTAATCGAAGACGAAGGATTGAAC
AGAGAAAAAGAAAATGATGTTTATCATTTTCAAAAATCACATAAAAAATCGTGATG
TTTATCGCAGTTATTTCTTTCTTTGTGTTTCGTTTCTAGTTGTATTTCTTTTCAGTGG
GGTTGGAATAAAAAGATTGATCAAGAAAAAAAAAAAAAAAAAAAAAAAAAAAA
AA
>Promoter of *L. vannamei* Vago3
CAAATATGCAAGCTTAAACACTCATGATGGGGAGTGTTTGCCCCGTTCCCGTGTGC
GTGTGTATGGCATATTTAATACGTTGTGTGTATTTGTGTGTTGTGTGTGTGTGTGT
GTGTATATGTCTACATGTTTATGTATATATGAATATACATTTATATACATGTAAATATAG

ATACTGATATACACATACACATATATTTGTACACACACACATATTTGTACACACAC
ATACACAGGAATTTTGTACACACACACATATATATATGTATGTATGTATATATATAAGC
ATGATGATGTTACACCATGCATTTGGGCAAAGAAAGAAACAAAGAGGAGAGAAA
GGGGGCGGGAGGAAGGGAGGAGCATAAGCGGCGGCAGGGATATAAAGGGCTCT
CGTGGCCACAGACTCCCAGGTCTCAGCAGCCCCGTCCTTAAGCACCACCAGCAAC
AAGTCACTATG

Primers:
PGL3- LvVago3-F: AAT*GGTACC*CAAATATGCAAGCTTAAACACTC
PGL3- LvVago3-R: AAT*AGATCT*AGTGACTTGTTGCTGGTGGT

>*Litopenaeus vannamei* Vago4 mRNA (Accession No. HQ541161.1)
GGAGAAGACAGCGCACACACCTCGCGTTCAGACACACACGCACACGCGACCCGA
TCTCAAGTGTATACTTCCGTAGCCGCCAAGTTCCGCCCGCTTGACAGGCTTCCGC
CCTCGACGTCGAGCTGGAGTAAAGATTCTTTGGGACTCCGCGTGCTTTTTTTTGCT
CCCGCTCTCGTCTTCTGGAGTGCGGGCGTGCGAGAGCGAGAGGGAAAAGGAAAA
CAGGTCACGGAGGAGGATCTCGACAGCGTCCGAGGCGAAAATGGGTTTTAGCGT
CGCGCAAGTCGCCCTGGTCCTGTGCCTGGCAGGAACCTCCCTCGCTGCGGTCGCC
ATCGGCCCTGCGGTCGTGCACCCAGACCACCCCGGGAAGTGCTGGCTGCCCAAG
CAGAGCCGGTCCTACGCCGACGGGGCCCAGTGGCAGGAGCCCAACTGCATGAGG
GCCACGTGTCTCTCGTACAAGTCCCAGCTCTACGTCGAGTATGCTACATGCGGTC
CGGTCGCAGCAGGACCCGGATGCAAGCCCGTGCAGGACCTGAGCCTCCCGTACC
CGAGCTGCTGCCCCATCATCTCCTGCCCGAACGCCGACCCCGATGCCCTGAAGGG
GGAGGAGTACGACGAGTTCACGAATTGGATCAGCGAGTACTACGACAACCCGAC
TCCGACGGCCTAAGGAGGGACTCGGCTGGAGAGTCCGGTTCTCGCGTCCGCACTT
GACCAAAGCTTTTATTTTATCTTTTTTTTTAATATCTTTTGTGTGTTCTTAGGCATT
TATATTTTCCCGAGAGAGTTCGCTTGCGTTTTGGCCTCGCTTTGTTAACTGGACTC
TTGAGGTAAGATGCCGTTGTGAAGATCAGTTGGAAATTTGAAATTATGATGCAGA
ATCTTTGGGAGACGAAGGAATCCAAGGCTAGCATTCCTTGGCAAAGGGAGCCAA
TGATTGATTTATTAAGATGTTTTTTTCCAAATGTATTTATTAAAAAAGAAAAAAA
AAAAAAAAAAAAA

>Promoter of *L. vannamei* Vago4
CAGTCGAGTTTCTCATAGGAGTCTATATCTGGCAACGCAAGAGATCCCTAATATG
GCTAGAAAATTAAAGATGCCAAAAAAATTAAGATAATACAGATAGTTGAGGATA
TTAACAACCCTGACGCTTCTTAACCCTCTCAACGCCAAAGCCCAACCACACAATC
TGCCCAATCTCCGTCGCTCTTGTAATGACACTTGCAAAAAGAGCGCACGCAGTGA
TCTTGACATTGAAACCTGACTGTGACGTCATCGTGTAAACAAGTTCCCGCGGCAT
ATAAAGGCTGGACCGAGAAGCCGCGCGAGGAGAAGACAGCGCACACACCTCGC
GTTCAGACACACACGCACACGCGACCCGATCTCAAGTGTATACTTCCGTAGTCGC
CAAGTTCCGCCCGCTTGACAGGCTTCCGCCCTCGACGTCGAGCTGGAGTAAAGAT
TCTTTGGGACTCCGCGTGCTTTTTTTTGCTCCCGCTCTCGTCTTCTGGAGTGCGGG
CGTGCGAGAGCGAGAGG**GAAAAGGAAAACA**GGTCACGGAGGAGGATCTCGACA
GCGTCCGAGGCGAAAATGGGTTTTAGCGTCGCGCAAGTCGCCCTGGTCCTGTG

GAAAAGGAAAACA：IRF1 binding motif

Primers:
pGL3-LvVago4-F: AAT*GGTACC*CAGTCGAGTTTCTCATAGGAGTCT
pGL3-LvVago4-R: TTC*AGATCT*CACAGGACCAGGGCGACTT

>*Litopenaeus vannamei* Vago5 (Accession No. HQ541162.1)
AACCCAGTGCAAGCCAGAACGCTCCATAGCCAGGCACGAAAGCTTGGTAGTTAT
AGACTTGACATTTTCCCTTTGGAGGAGGACCCGGTATAAGGATGGCCAGAGGGA
TTGTGATGCTGCTTGTGCTGACGGTCGCTGCTACGGTGACCCAAGGCGCTCTCCA
ACATCTGATCGCAGATAATCCAGAGTACCCAGGGATGTGCGCCGACAAGAAGGC
AGGCATTTTCCCTATGGGCGCAACATGGTTTTGGAAGGCTGTGTACGGGCACAC
TGCAGCCGTTCTGAGGGCAGGATGGTTATCACGTATGCCTCCTGTTCTCCGTTCG
GCCTTCCCCCAAACTGTGAACTGGTTACAGACGAGTCCTTGGTTTACCCTGGTTG
CTGCCCGAAGCCTAAATGCTCCTAAGAAATTCTGAGTTGTACCCCAATGACTGCT
GTTATCCTTACTAATTCATACCTTATTTAGTGAAGCTCCTTTACATATACCTCCAT
TAATATGTTAAACACATGTTCAGCATTGTATCATGGATGAAGGCAATATATATGA
AATGTATAATATTGATATTGGGGAAGAAAAAAAACATTATAAAGAGTTAAAAAA
AAAAAAAAAAAAAAAAAAAAAAA

>Promoter of *L. vannamei* Vago5
GGTAAATTAGCCCTTTTACCAATTAATCATCAATTGAGTTCAACGCTACTTTGATA
CAAAAAAAAGGTATTACATTAGGTATCATTACATTAACGAAGGCCTTTAGGACA
CATACCCACACCAAGAAAACAAGTGCGAGGCGCGCGATAAGCTGGGGAATTCTC
ACTCCCAGAGCAAAAAAACATCCTCTGATATCACGCAATCTTCAATGTGCTTGCT
CTATATAAGAGTGCGACTGCACATACCCGCAACCCAGTGCAAGCCAGAACGCTC
TATAGTCAGGCACGAAAGCTTGGTAGTTATAGACTTGACATTTTCCCTTTGGAGG
AGGACCCGGTATAAGGATGGCC

Primers:
pGL3-LvVago5-F:AAT*GGTACC*GGTAAATTAGCCCTTTTACCAAT
pGL3-LvVago5-R:TTC*AGATCT*CTCCTCCAAAGGGAAAATG

Type-I-IFN
>IFN-α mRNA (Accession No. NM_024013.2)
AGAACCTAGAGCCCAAGGTTCAGAGTCACCCATCTCAGCAAGCCCAGAAGTATCT
GCAATATCTACGATGGCCTCGCCCTTTGCTTTACTGATGGTCCTGGTGGTGCTCAGC
TGCAAGTCAAGCTGCTCTCTGGGCTGTGATCTCCCTGAGACCCACAGCCTGGATA
ACAGGAGGACCTTGATGCTCCTGGCACAAATGAGCAGAATCTCTCCTTCCTCCTG
TCTGATGGACAGACATGACTTTGGATTTCCCCAGGAGGAGTTTGATGGCAACCAG
TTCCAGAAGGCTCCAGCCATCTCTGTCCTCCATGAGCTGATCCAGCAGATCTTCAA
CCTCTTTACCACAAAAGATTCATCTGCTGCTTGGGATGAGGACCTCCTAGACAAAT
TCTGCACCGAACTCTACCAGCAGCTGAATGACTTGGAAGCCTGTGTGATGCAGGA

GGAGAGGGTGGGAGAAACTCCCCTGATGAATGCGGACTCCATCTTGGCTGTGAAG
AAATACTTCCGAAGAATCACTCTCTATCTGACAGAGAAGAAATACAGCCCTTGTGC
CTGGGAGGTTGTCAGAGCAGAAATCATGAGATCCCTCTCTTTATCAACAAACTTGC
AAGAAAGATTAAGGAGGAAGGAATAACATCTGGTCCAACATGAAAACAATTCTTA
TTGACTCATACACCAGGTCACGCTTTCATGAATTCTGTCATTTCAAAGACTCTCAC
CCCTGCTATAACTATGACCATGCTGATAAACTGATTTATCTATTTAAATATTTATTTAA
CTATTCATAAGATTTAAATTATTTTTGTTCATATAACGTCATGTGCACCTTTACACTG
TGGTTAGTGTAATAAAACATGTTCCTTATATTTACTC

>Promoter of IFN-α

GGCCTCATGTCAACTGAAGCCTGACTGGCCAGTGTCTCAAAGTCACAGATGATGA
CCTGATCCCTCAGGAACAGATGGTGTTTCAGCTTTGTGGGAGTGACTGTCAAGGT
ATGGAGCACTTAGGTGGTCTTTGAAACCTGTCAGGTTTCACATCTCTGCTTTGAGT
GAAAAGCTCATCACCCACAGTAGTGAGGAATGTGCCTATACTTGCTGGGGCTGATC
TGTTGAAACTTTATGTGTAGACAATGGAAACATCCAGGAGCATTTCTGCTTTCATG
TAGCCTCTTAATAATTGATGTCCCTGAAGTCCTGTGTCCTCAGGGACAGTTCCTCT
GATTCCTGGATTGTACCAGCTTTCATGATGTTAAATCTAATCTGTAAAACTCTGAGC
ATTAATCTCCATGAAAATAAGACCTTGTTCTTTCTCATTTAAATGCCCCTTTTTTTTC
TTGTCTAATATTCTGAGTAGGATTTCCAGTACTGTGTTGAAAGAAGTAGTGAGAGT
GGGCATCCTTATCTTATAATAAATCTCAGAAAAAAGATTCCAACATTTCACCACTGA
CTATAGTGTTAGCTAATGGCTTATCTTATAGCTAATAAGGAATGTGTCTCTTTATTCT
GAGGTATATTCTTTATATACCTAATTTGTTATAAATTGTATTAGAGATGGATTTTAAAT
TTTGTCAAAATAATTTTAGGCATGCATAAAAATAGTTATGATTTTTAATCTTTTATTG
TGTAAATAAGGAGTATGGCATTTATTGATTCCAACATATTAAAATATTCTTGCATCCC
AGGAATAAATCAAGCTTGATCATAATAAATGATCCTTTTATAGTGCTTTTGAATTTCA
TTTGCAACTACTTTGTGGATGATTTTGCATCTGTGTTCATCAGGAATATTGGCTGTA
ATTTTTTTTTTTCTTGTAATGTCCATCTCTGGCTTAGGTATCAAGGTAATGCTGGCTT
CATAAAATGAGTTTGGGAGTATTCCTCCTTAATTTTTTCAAAGATTTGGTTCTGTTT
AAATGTTTGGTGAAATTCAGGAGTAATCAGATCTAAAAAAACGTAAGAACACTAA
AAATAAATGATAAAAATATTTTAAAGTTTTAAGTGTTTATCACTTTCAAATAATTTTT
AAGTATTCATTACTTAGAAAATGTTTATCTATAAACATTAGAAAATGTTTATAATTTG
TTAATTTGCTTATGTAATTTGATACACTACAATTTATTTGTTTGTTTGTTTTTTGAAA
CAGAGTCTCACTCTGACACCCAGGCTGGAGTGCAGTGGCACAATCTCGGCTCACT
GCAACCTCCACCTCATGAGTTCAAGGAATTCCCTGCCTCGGCCTCCTGAGTAGCTG
GGATTACAGGTGCACACAACCATGCCTAGCTAATTTTTTTTTTGTTTTTTTTTTGTTT
TTTTCGTTGAGATGGGGTTTCACCGTCTTGGCCAGGCTGGTCTTGAACTCCTGACC
TCGTGATCCACCCACCTCGGACTCCCAAAGTGCTGGGATTACAGGCGTGAGCCAC
CTCGCCAGGCCTACAGTACAATTTATATCAGTAAATAAATTACGTTGAAGTCCATAA
GCAAATTTAGTTATAAAGTTAAGAAAAATTTGAATAGATTTTAAATTTAACTTTTAG
CTTAAATTTTTCATTTGGTTATTTTTAAACCTGCATTGAATACAAAGATTAAACTTTG
TACTTTTTGATTATAGAGATATACATATAGTATATAAATAGATACATATTGTATCTGTG
TTATTAAATTTTCACGGTGGGTTCAATTAGGAAAAAGATATCTAAAAAGTCTCTGG
GAACAAGATGGGGAAGACAATAATGAAAAACAAAACATTTGAGAAACACGGCTC
TAAACTCATGTAAAGAGTGCATGAAGGAAAGCAAAAACAGAAATGGAAAGTGGC
CCAGAAGCATTAAGAAAGTGGAAATCAGTATGTTCCCTATTTAAGGCATTTGCAGG

AAGCAAGGCCTTCAGAGAACCTAGAGCCCAAGGTTCAGAGTCACCCATCTCAGC
AAGCCCAGAAGTATCTGCAATATCTACGATGGCCTCGCCCTTTGCTTTACTGATGGT
CCTGGTGGTGCTCAGCTGCAAGTCAAGCTGCTCTCTGGGCTGTGATCTC

Primers:
pGL3-IFN-α-KpnI-F: GG*GGTACC*TCATGTCAACTGAAGCCTGACTGG
pGL3-IFN-α-XhoI-R: CCG*CTCGAG*ATACTTCTGGGCTTGCTGAGATGG

>IFN-β mRNA (Accession No. NP_002167.2)
ACATTCTAACTGCAACCTTTCGAAGCCTTTGCTCTGGCACAACAGGTAGTAGGCG
ACACTGTTCGTGTTGTCAACATGACCAACAAGTGTCTCCTCCAAATTGCTCTCCTG
TTGTGCTTCTCCACTACAGCTCTTTCCATGAGCTACAACTTGCTTGGATTCCTACAA
AGAAGCAGCAATTTTCAGTGTCAGAAGCTCCTGTGGCAATTGAATGGGAGGCTTG
AATACTGCCTCAAGGACAGGATGAACTTTGACATCCCTGAGGAGATTAAGCAGCT
GCAGCAGTTCCAGAAGGAGGACGCCGCATTGACCATCTATGAGATGCTCCAGAAC
ATCTTTGCTATTTTCAGACAAGATTCATCTAGCACTGGCTGGAATGAGACTATTGTT
GAGAACCTCCTGGCTAATGTCTATCATCAGATAAACCATCTGAAGACAGTCCTGGA
AGAAAAACTGGAGAAAGAAGATTTCACCAGGGGAAAACTCATGAGCAGTCTGCA
CCTGAAAAGATATTATGGGAGGATTCTGCATTACCTGAAGGCCAAGGAGTACAGTC
ACTGTGCCTGGACCATAGTCAGAGTGGAAATCCTAAGGAACTTTTACTTCATTAAC
AGACTTACAGGTTACCTCCGAAACTGAAGATCTCCTAGCCTGTGCCTCTGGGACT
GGACAATTGCTTCAAGCATTCTTCAACCAGCAGATGCTGTTTAAGTGACTGATGGC
TAATGTACTGCATATGAAAGGACACTAGAAGATTTTGAAATTTTTATTAAATTATGA
GTTATTTTTATTTATTTAAATTTTATTTTGGAAAATAAATTATTTTTGGTGCAAAAGTC
A
>Promoter of IFN-β
TAGTGGCCTAATAACCCTATTTCCCAGACCTCTTCTCATTACAAGGAAAAACTCATA
TGCAGATAGTTCTAAAGGTATGAAGTGAAAAGATAAAGATTTTTCTTCCTTGCTGC
ATCCTCACCCCATCAGCATTATTCCCCAGGGTAACTACTATTAATAGATAGTAATTCT
ACCCAAAGGAAAAAATCATATGCATATAACAGCATCATATGTATACCTTTCTAGTAA
CTTACAAAACAAATGATAATATCATATCCTTTCTTATGTGTATTGCTCTTTTCACTAA
ATGTATCTGTGATATGTGTCTATATCAGCTGATTGTCCTTTTTGATGGCTGAATAATA
TTCCATCTTGTCCACGTGATAGTATTACTTGACAAGCTCCCTGCTGATGGACATTTG
TCTTTGTTACTATGATAGTAATATAATCAACATTTATATATGTTTTGTATGTATCTATAA
TACACATGCACATACACATGCATATTTCTGCAGGGATAGCCATAGTAAATAACTAGT
AACGGTATTGCAAGTTAAAGGAACAATCTCATTGCTTGAAATTTTAAATTTTGAAA
TACACTGCCAATTTTCATGGTCTCTCCTTGTAAGCTAGTTTGGGCTTTCTCACAGCA
TGACAGGCTCAGGGCAGTCAGACCATCCTGGCCAAAGAGCAGAGTGCCACAGAC
CACAACTGCTTCTAATCAGCCATCTTCCCAAAGCCTTCTCTTTTTTCTATTAATAAC
TTTGTATGAGATTCCATCTTAATACTTTTCTGTTGTTTGGTCTTGTAAGAGCTTATTT
TTCCTGAACCAGGAAGTGGTTCAGGGCGGTTTTTCTAACTTCACAGAGCTCCCTC
TTCTGTTAGCTTTTGTGAAATGGTCAAAAACATAGCAGCCTGCCTTCTGAGTTCTC
CATCCCACCCTGGTTGGGCCTTCTCTATCCTTGTCTGTGTTGTTTATATCCTGCTGA
AGTGTGATTCCACTTGTGGCAGTTTCTCCTCTGTGTAGGATCAAAAGGGCTGTGAC

TGGTTGGTTTGAAAATTTCTTATACCCTAGACTATTCCAGTGCCTTTCAGAAGTTTC
CAAGGCCCTCTCACACTAATCTATTATCATATTGGGCAAAACTCCTTGCAGTTTCAG
CTACTATTCCCTGATTGACTTTTCAGTAAATCTATCTCTCAGTCTTTCAGTATCCAAA
GAAGATTGGTTCTAGGACCACCATCCCGCTGCCTCCACAGATACCAAAATCAGAG
GATGCTCAATTCCCTCTTATAAAACGTTGCAGTATTTGCATATAATCTGCACATGTAT
TTCTGTATATTTTAAATCATCCCTAGATTACTTATAATACCTGATACAATATAAATGCT
AAATAGCTGTAACACTGTATCTTTAAAATTTACATTATTTTTTGTTGTTGTATTATTAT
TTTTATTGTATTTTTAAAAAATATTTCCATCTACAGTCAGTAGAATCCACGGATACA
GAACCTATGGATAGGAAGGACCAACTGTATCTTTTAGTGTTTTGAGGTTCTTGAAT
TCTCAGGTCGTTTGCTTTCCTTTGCTTTCTCCCAAGTCTTGTTTTACAATTTGCTTT
AGTCATTCACTGAAACTTTAAAAAACATTAGAAAACCTCACAGTTTGTAAATCTTT
TTCCCTATTATATATATCATAAGATAGGAGCTTAAATAAAGAGTTTTAGAAACTACTA
AAATGTAAATGACATAGGAAAACTGAAAGGGAGAAGTGAAAGTGGGAAATTCCT
CTGAATAGAGAGAGGACCATCTCATATAAATAGGCCATACCCATGGAGAAAGGACA
TTCTAACTGCAACCTTTCGAAGCCTTTGCTCTGGCACAACAGGTAGTAGGCGACA
CTGTTCGTGTTGTCAACATGACCAACAAGTGTCTCCTCCAAATTGCTCTCCTGTTG
TGCTTCTCCACTACAGCTCTTTCCATGAGCTACAACTTGCTTGGATTCCTACAAAG
AAGCAGCAATTTTCAGTGTCAGAAGCTCCTGTGGCAATTGAATGGGA

Primers:
pGL3-IFN-β-KpnI-F: GG*GGTACC*CCCAGACCTCTTCTCATTACAAGG
pGL3-IFN-β-XhoI-R: CCG*CTCGAG*AAGTTGTAGCTCATGGAAAGAGCTG

>IFN-ω mRNA (Accession No. NM_002177.1)
GATCTGGTAAACCTGAAGCAAATATAGAAACCTATAGGGCCTGACTTCCTACATAA
AGTAAGGAGGGTAAAAATGGAGGCTAGAATAAGGGTTAAAATTTTGCTTCTAGAA
CAGAGAAAATGATTTTTTTCATATATATATGAATATATATTATATATACACATATATACA
TATATTCACTATAGTGTGTATACATAAATATATAATATATATATTGTTAGTGTAGTGTGT
GTCTGATTATTTACATGCATATAGTATATACACTTATGACTTTAGTACCCAGACGTTT
TTCATTTGATTAAGCATTCATTTGTATTGACACAGCTGAAGTTTACTGGAGTTTAGC
TGAAGTCTAATGCAAAATTAATAGATTGTTGTCATCCTCTTAAGGTCATAGGGAGA
ACACACAAATGAAAACAGTAAAAGAAACTGAAAGTACAGAGAAATGTTCAGAAA
ATGAAAACCATGTGTTTCCTATTAAAAGCCATGCATACAAGCAATGTCTTCAGAAA
ACCTAGGGTCCAAGGTTAAGCCATATCCCAGCTCAGTAAAGCCAGGAGCATCCTC
ATTTCCCAATGGCCCTCCTGTTCCCTCTACTGGCAGCCCTAGTGATGACCAGCTATA
GCCCTGTTGGATCTCTGGGCTGTGATCTGCCTCAGAACCATGGCCTACTTAGCAGG
AACACCTTGGTGCTTCTGCACCAAATGAGGAGAATCTCCCCTTTCTTGTGTCTCAA
GGACAGAAGAGACTTCAGGTTCCCCCAGGAGATGGTAAAAGGGAGCCAGTTGCA
GAAGGCCCATGTCATGTCTGTCCTCCATGAGATGCTGCAGCAGATCTTCAGCCTCT
TCCACACAGAGCGCTCCTCTGCTGCCTGGAACATGACCCTCCTAGACCAACTCCA
CACTGGACTTCATCAGCAACTGCAACACCTGGAGACCTGCTTGCTGCAGGTAGTG
GGAGAAGGAGAATCTGCTGGGGCAATTAGCAGCCCTGCACTGACCTTGAGGAGG
TACTTCCAGGGAATCCGTGTCTACCTGAAAGAGAAGAAATACAGCGACTGTGCCT
GGGAAGTTGTCAGAATGGAAATCATGAAATCCTTGTTCTTATCAACAAACATGCAA

GAAAGACTGAGAAGTAAAGATAGAGACCTGGGCTCATCTTGAAATGATTCTCATT
GATTAATTTGCCATATAACACTTGCACATGTGACTCTGGTCAATTCAAAAGACTCTT
ATTTCGGCTTTAATCACAGAATTGACTGAATTAGTTCTGCAAATACTTTGTCGGTAT
ATTAAGCCAGTATATGTTAAAAAGACTTAGGTTCAGGGGCATCAGTCCCTAAGATG
TTATTTATTTTTACTCATTTATTTATTCTTACATTTTATCATATTTATACTATTTATATTCT
TATATAACAAATGTTTGCCTTTACATTGTATTAAGATAACAAAACATGTTCAGCTTTC
CATTTGGTTAAATATTGTATTTTGTTATTTATTAAATTATTTTCAAAC

>Promoter of IFN-ω
TCTGCACAGCAAAAGAAACTACCATCAGAGTGAACAGGCAACCTACAGAATGGG
AGAAAATTTTTGCAATCTACTCATATGACAAAGGGCTAATATCCAGAATCTACAATG
AACTCAAACAAAGTTACAAGAAAAAAACAAACAACCCCATCAAAAAGTGGGCGA
AGGATATGAACAGACACTTCTCAAAAGAAGACATTTATGCAGCCAAAAGACACAT
GAAAAAATGCTCATCATCACTGGCCATCAGAGAAATGCAAATCAAAACCACAATG
AGATACCATCTCACACCAGTTAGAATGGCGATCATCAACAAGTCAGGAAACAACA
GGTGCTGGAGAGGATGTGGAGAAACAGGAACACTTTTACACTGTTGGTGGGACT
GTAAACTAGTTCGACCATTGTGGAAGTCAGCGTGGCGATTCCTCAGGGATCTAGA
ACTAGAAATACCATTTGACCCAGCCATCCCATTACTGGGTATATACCCAAAGGATTA
CAAATCATGCTGCTATAAAGACACATGCACACGTATGTTTATTGCGGGACTATTCAC
AATAGCAAAGACTTGGAACCAACCCAAATGTCCAACAACGATAGACTGGATCTAG
AAAATTTGGCACATATAAGCTTCTAATGAAAGACAACATCATCATTGATCCCCTGTG
CCCACCTGAGAAACACTGAGGATAACAAATGTTCAATAGCTTACGTAAAAGAATT
GAACAATAAAAACCGTGTCAGGATTCCGAGCTGGGATACTGACACAGCCAGTGGA
GCACTATTGACCTCAGTAAGCAAATGGTGGAAAATTATTTGACTAATGAAGGTAAA
ATGATTATAATTGGGTAATCCTTCCACCTTCCTTAGACAAAAGAAACCTTTGCTATC
AGTACCTGAGGGCTGTCGAAACTTTTGATACCTGGACACATGGGTCAGGAGGAAA
GGAGGTCGCCACTGAGAGCCAGGGTCCTCCAGACAAACACTCTAGGGGCACCGG
CAAGGGTCTTGGAGGGTTAGTTGCTTTGCTCACAAAGTAACCACTACCCTCTCAC
CAAAAGAGTTCTGAAGTTATCAGGATGTTGTGAGACTGCTCCCTGGGAGCGAGTC
TGGAAGAGTTTGGGTTGAGAGTATTTGTTACTCTAGAGGAAAAGGTATTACTAGAT
GTGATAACTACCTTGTGTGTGAGGAGACGCTGGGAAAGTAAAATATCTGCGAATTA
GAATCTCTACATCCATATGCGCTTGTAAAGAATCAGGATTCCAGCTGGGATATTGAC
AGATAGGGGAGCACTATTGACCTCAGTAAGCAAATGATGGAAAGAAAAAGCAGT
GTCTATATTAATCTTCACCTTGTGGTCCCAGTGAAGCGTAACCACATGGACCTGCAT
GAATGTTTTCTAGAAGGAAGAAAAATGTCAGAGAAGCTCATTCGCTAATCATCATC
ATCCTCTGTTGTGAATAAAACAGCAAACAAAACTGCGTTTTTGGCTCATATCTGTA
ATTCCAACTACTTGGGAGGCTGAGGCAGGAGGCCTCAGCTTCAGGCCAGGAGTTT
GAGACCAGCCTGAACAACATAGTGGGACCTCATCTCTAACAACAACAACAACAA
AAAAATTAGCCAGGCATGATGGTGCACACCTGTTGTCCCAGGTACTCAGGGGGCT
GAGGCACAGAGATTGCTTGAGCCCAGGAGTTAAAGGCTGCAGTGACCTCTGATCA
TGCCACCACACTCCAACCTGGGTGACGGATTAAGACCCCATCTCTAAAAAACAAA
CAAACAACAGCAAAATGCTGTTCTTGGTTCTAAAGAGCTTATTTGCTGCAGATGAT
CTGGTAAACCTGAAGCAAATATAGAAACCTATAGGGCCTGACTTCCTACATAAAGT
AAGGAGGGTAAAAATGGAGGCTAGAATAAGGGTTAAAATTTTGCTTCTAGAACAG

AGAAAATGATTTTTTTCATATATATATGAATATATATTATATATAC

Primers:
pGL3-IFN-ω-KpnI-F: GG*GGTACC*ATCAGAGTGAACAGGCAACCTACAG
pGL3-IFN-ω-XhoI-R: CCG*CTCGAG*TTTTAACCCTTATTCTAGCCTCCAT

Type-III-IFN
>IL28A mRNA (Accession No. BC113583.1)
CCCTGGGTGACAGCCTCAGAGTGTTTCTTCTGCTGACAAAGACCAGAGATCAGGA
ATGAAACTAGACATGACTGGGGACTGCACGCCAGTGCTGGTGCTGATGGCCGCAG
TGCTGACCGTGACTGGAGCAGTTCCTGTCGCCAGGCTCCACGGGGCTCTCCCGGA
TGCAAGGGGCTGCCACATAGCCCAGTTCAAGTCCCTGTCTCCACAGGAGCTGCAG
GCCTTTAAGAGGGCCAAAGATGCCTTAGAAGAGTCGCTTCTGCTGAAGGACTGCA
GGTGCCACTCCCGCCTCTTCCCCAGGACCTGGGACCTGAGGCAGCTGCAGGTGA
GGGAGCGCCCCATGGCTTTGGAGGCTGAGCTGGCCCTGACGCTGAAGGTTCTGG
AGGCCACCGCTGACACTGACCCAGCCCTGGTGGACGTCTTGGACCAGCCCCTTCA
CACCCTGCACCATATCCTCTCCCAGTTCCGGGCCTGTATCCAGCCTCAGCCCACGG
CAGGGCCCAGGACCCGGGGCCGCCTCCACCATTGGCTGTACCGGCTCCAGGAGG
CCCCAAAAAAGGAGTCCCCTGGCTGCCTCGAGGCCTCTGTCACCTTCAACCTCTT
CCGCCTCCTCACGCGAGACCTGAATTGTGTTGCCAGTGGGGACCTGTGTGTCTGA
CCCTCCCACCAGTCATGCAA
>Promoter of IL28A
CTCCAAATCTGTGCATTAGCAATCTGCGTTGCTGAATGCACCCCTCCTTGCCAAGG
TCACACGGCTAGTGAGGGTCAGAACCAGGTTTGAACCCCAGACCCTTCATCTCCA
AGACCCATGCTCTTCACCACTGCCTGAACTTCCCTAAGAAAGACGGCACCCACGT
GGTGTCCTTCAAGTCCTTCGTCACACCTCAGTTCTTGAGCAGAGCCTCATATTCCT
GAGTCCTTCCTTGCCTGGGCAATTAAGAAATATTGGCCTTGGCTGGGCTCAGAGGC
TCATGCCTGTAATCCCAGCACTTTGGGAGGACGAGGAAGGTGGATCATGAGGTCA
GGAGTTCAAGACTAGCCTGGCCAGCATGGTGAAACCCTGTCTCTACTAAAAATAC
AGAAATTAGCTGGGCATGGTGGCACATGCCTGTAATCCAAGCTACTCAAGAGGCT
GAGGCAGGAGAATCACTTGAACCCAGGAGGCAGAGGTTGCAGTGAGCTGAGATG
GTGCCTCTGCACTCCAGCCTGGGCAACAGAGCGAGACTCCATCTCTCTTGCTCAC
TCACTCTCTCTCATGCTCTCTCTCTCTCTCTCTCTCTCTCTCTCTCTCTATATATATAT
ATATATATATATATACACACACATATATGCCTCTGGCCGGGCGCGGTGGCTCACGCCT
ATAATCCCAGAACTTTGGGAGGCCGAAGAAGGCAGATCACGAGGTCAGGAGTTC
AAGACCAACCTGGCCAATATGGCAAAACCCCGTCTCTACTAAAAATACAAAAATT
AGTTGGGCGTGGTGGTGCATGCCTATAGTCCTTTCTACTCGGGAGGCTGAAGCAG
AAGAATCCCTTGAACCAAGGAGGTGGAGGTTGCAGTGAGTCGAGATCACGCTGC
TGCACTCCAGCCTGGGCTACAGAGCAAGACTCCATCTCAAAAAAAAAAGAAAAA
AGAAAGAAAGAAAGAAATCATGGCCTCTGGGCACAGTGGCTCATGCCTGCAACC
CCAGCAATTTGGGAGGCCAAGACAGACAGATCACTTGACGTCAAGAGTTCGAGA
CCAGCCTGGCCAATTGGTGAACTGTCATCTCTACTAAAACCATAAAAATTAGCTGG
GAATGGTGGCACAAATCTGTAATCTCAGCTACTTGGGAGGCTAAGGCAAGAGAAT
CGCTTGAACCCAGGAGGTGGAGGTTGCAGTTAGTCAAGATTTTGCACTGCACTCC

AGCCTGGGTGACCGAACAAGACCCTGTCTCAAAATATATATATATATATATGCCAGG
AGGGGTGGCTCAGGCCTGTAATCTCAGCACTTTAATAGGCTGGGTGAGGAGGATG
GCTTGAGCCCAGGAGTTTGAGGCTGCAGTGAGCTGTGATCATGCCATTGCACTCC
AGTGACAGAGTGAGACCCTGTCTTAAACAACAACAAAACCAGAGCAGGTGGAAT
CCTCCTGGGAACATACCTTCCTGTAGGTTACCCCTGAGTCTCCATCAGTTTCTCTTT
CCCTCCAGCTGCTCATCTGGCTCACTAGCCCTGCCCTGCTCTGGGCTTTCCCAGCC
TGGGGCTCCCCTGGTGGCCGGTGTCTTACCTGAGGCTGTGTTTTCACTTTTCCTAC
ATCAGCTGGGACTGCCCTTCTGTCAGGGATAAAAGCTGCCCCATGGAGCTCAGGC
AGGAATTACATCCCAGACAGAGCTCAAAACTGACAGAAAGAGTCAAAGCCAGGA
CACAGTCTGAGATCCAGAAGAGGGGACTGAAAAGAACAGAGACTCCAGACAAG
ACCCAAACAGACCCTGGGTGACAGCCTCAGAGTGTTTCTTCTGCTGACAAAGACC
AGAGATCAGGAATGAAACTAGGTGAGTCCCACATCTCTGTCCGTGCTCAGCTCCT
GCAGCCCCTGCCCTCAGTGGGCAGCCTCTCCATCCCCTCAGCTCCCTTTCTCTCTG
TGACACAG

Primers:
pGL3-IL28A-KpnI-F: GGGGTACCGCAATCTGCGTTGCTGAATGC
pGL3-IL28A-XhoI-R: CCGCTCGAGCCTGATCTCTGGTCTTTGTCAGC

>IL28B mRNA (Accession No. NP_742151.2)
AGACATGACCGGGGACTGCATGCCAGTGCTGGTGCTGATGGCCGCAGTGCTGACC
GTGACTGGAGCAGTTCCTGTCGCCAGGCTCCGCGGGGCTCTCCCGGATGCAAGGG
GCTGCCACATAGCCCAGTTCAAGTCCCTGTCTCCACAGGAGCTGCAGGCCTTTAA
GAGGGCCAAAGATGCCTTAGAAGAGTCGCTTCTGCTGAAGGACTGCAAGTGCCG
CTCCCGCCTCTTCCCCAGGACCTGGGACCTGAGGCAGCTGCAGGTGAGGGAGCG
CCCCGTGGCTTTGGAGGCTGAGCTGGCCCTGACGCTGAAGGTTCTGGAGGCCACC
GCTGACACTGACCCAGCCCTGGGGGATGTCTTGGACCAGCCCCTTCACACCCTGC
ACCATATCCTCTCCCAGCTCCGGGCCTGTATCCAGCCTCAGCCCACGGCAGGGCCC
AGGACCCGGGGCCGCCTCCACCATTGGCTGCACCGGCTCCAGGAGGCCCCAAAA
AAGGAGTCCCCTGGCTGCCTCGAGGCCTCTGTCACCTTCAACCTCTTCCGCCTCCT
CACGCGAGACCTGAATTGTGTTGCCAGCGGGGACCTGTGTGTCTGA
>Promoter of IL28B
TACTTGCCTTGTGGAGGAGTAGTGTCCGTTGAGATCACGTTTTAAACTCTCCAGCT
GAGGTCCTAGTATGGTCTTAATGAGTGGATTCTATTAAATAATCACTCACATAAATA
CACAAACAATTGTGTTATTAATTTTTTCTCCAAATCTGTGCATTAGCAATCTGCGTT
GCTGAATGCACCCCTCCTTGCCAAGGTCACACGGCTAGTGAGGGTCAGAACCAGG
TTTGAACCCCAGACCCTTCATCTCCAAGACCCATGCTCTTCACCACTGCCCGAACT
TCCCTAAGAAAGACGGCACCCACGTGGTGTCCTTCAAGTCCTTCGTCACACCTCA
ATTCTTGAGCAGAGCCTCATATTCCTGAGTCCTTCCTTGCCTGGGCAATTAAGAAAT
ATTGGCCTCTGGGCATGGTGGCTCACACTGAAATCCCAGCAATTTGGGAGGCCTA
GACAGAGAGATGACTTGACATCAGGAATTTGAGACCAGCCTTGCCAACATGGTGA
AACGCCATCTCTACTAAAAATATAAAAATTAGCTGGGAATGGTGGCACAAATCTGT
AATCTCAGCTACTTGGGAGGCTAAGGCAAGAGAATTGCTTGAACCCAGGAGGCG
GAGGTTGCAGTTAGCCAAGATTTTGCACTGCACTCCAGCCTGGGTGACCGAACAA

GACCCTGTCTCAAAATATATATATATATATATATATATATGCCAGGAGTGGTGGCTCAG
GCCTGTAATCTCAGCACTTTAATAGGCTGGGTGAGGAGGATGGCTTGAGCCCAGG
AGTTTGAGGCTGCAGTGAGCTGTGATCATGCCATTGCACTGCAGTGACAGAGTGA
GACCCTGTCTTAAACAACAACAAAACCAGAGCAGGTGGAATCCTCTTGGGAACAT
ACCTTCCTGTAGGTTACCCCTGAGTCTCCATCAGTTTCTCTTTCCCTCCAGCTGCTC
ATCTGGCTCACTAGCCCTGCCCTGCTCTGGGCTTTCCCAGCCTGGGGCTCCCCTGG
TGGCCGGTGTCTTACCTGAGGCTGTGTTTTCACTTTTCCTACATCAGCTGGGACTG
CCCTTCTGTCAGGGATAAAAGCTGCCCCATGGAGCTCAGGCAGGAATTACATCCC
AGACAGAGCTCAAAACTGACAGAAAGAGTCAAAGCCAGGACACAGTCTGAGATC
CAGAAGAGGGGACTGAAAAGAACAGAGACTCCAGACAAGACCCAAACAGACCC
TGGGTGACAGCCTCAGAGTGTTTCTTCTGCTGACAAAGACCAGAGATCAGGAATG
AAACTAGGTGAGTCCCACATCTCTGTCCGTGCTCAGCTCCTGCAGCCCCTGCCCTC
AGTGGGCAGCCTCTGCATTCCCTCAGCTCCCTTTCTCTCTGTGACACAGACATGAC
CGGGGACTGCATGCCAGTGCTGGTGCTGATGGCCGCAGTGCTGACCGTGACTGGA
GCAGTTCCTGTCGCCAGGCTCCGCGGGGCTCTCCCGGATGCAAGGGGCTGCCACA
TAGCCCAGTTCAAGTCCCTGTCTCCACAGGAGCTGCAGGCCTTTAAGAGGGCCAA
AGATGCCTTAGTGAGTCTCCCCCTGCCCTCCTGCCATGGACTAGCCTCCACCCGCA
CTCCAAGGGTC

Primers:
pGL3-IL28B-KpnI-F: GG*GGTACC*AGGTCCTAGTATGGTCTTAATGAGTG
pGL3-IL28B-XhoI-R: CCG*CTCGAG*GACTTGAACTGGGCTATGTGGC

>IL29 mRNA (Accession No. AAI26184.1)
TAAAAAGCAGAGCCATGCCGCTGGGGAAGCAGTTGCGATTTAGCCATGGCTGCAG
CTTGGACCGTGGTGCTGGTGACTTTGGTGCTAGGCTTGGCCGTGGCAGGCCCTGT
CCCCACTTCCAAGCCCACCACAACTGGGAAGGGCTGCCACATTGGCAGGTTCAAA
TCTCTGTCACCACAGGAGCTAGCGAGCTTCAAGAAGGCCAGGGACGCCTTGGAA
GAGTCACTCAAGCTGAAAAACTGGAGTTGCAGCTCTCCTGTCTTCCCCGGGAATT
GGGACCTGAGGCTTCTCCAGGTGAGGGAGCGCCCTGTGGCCTTGGAGGCTGAGC
TGGCCCTGACGCTGAAGGTCCTGGAGGCCGCTGCTGGCCCAGCCCTGGAGGACG
TCCTAGACCAGCCCCTTCACACCCTGCACCACATCCTCTCCCAGCTCCAGGCCTGT
ATCCAGCCTCAGCCCACAGCAGGGCCCAGGCCCCGGGGCCGCCTCCACCACTGG
CTGCACCGGCTCCAGGAGGCCCCCAAAAAGGAGTCCGCTGGCTGCCTGGAGGCA
TCTGTCACCTTCAACCTCTTCCGCCTCCTCACGCGAGACCTCAAATATGTGGCCGA
TGGGGACCTGTGTCTGAGAACGTCAACCCACCCTGAGTCCACCTGACACCCCACA
CCTTATTTATGCGCTGAGCCCTACTCCT
>Promoter of IL29
GCGTGAGCCACCATGCCCAGCTGCACATCCCAGCATTCAAAGACCAAAGAGCCTG
GAGTTCTGATGTTTAAGGGCAGGTGCAGGGTGTCCCAGCTCATAAGAGAGAGCAA
ATTCTCCTTTCCTCTGCCTTTTTGTTCTATTCAGGCCCTCGGCCAATTGGATGGTGC
CCACCACATTGGGTAACAACGGGTCTTCCTTACTCAGTCCATTGATTCAAATGCCA
ATCTCTTCTGGAAACACCCCAGAGTCATACCCAGAATTAACGCATCACCAGCTATC
TGATAAACTTAACCAGTCAAGGTGACACCTAAAATTAACCATCACAATTATAAAAA

TAACTACTCAGAGAAACATTAGGAGCATGAACTGAAATTAGTTAATGGGACATTCT
TAAACCAATGGCAGAAGCTCCTTCTTGGCCAGGAGCAGTGGCTCATGCCTTTAATA
CTAGCACTTTGCGAGGCTGAAGCAGGAGGATGGCTTAAGGCCAGGAGTTCAAGA
CTGGCCTGGGCAACATAGTGAGACCCCTATCTCTACAAAAATAAATAAATAAATAA
TAAAGTAAGGTGGTGGCTCACGCCTGTAATCCCAGCACTTTGGGAGGCCAAGGCA
GGCAGATCATCTGAAGTCAGGAGTTCGAAGCCAGCGTGACCAACATAGTAAAACC
CAGTCTCTACTAAAAATACAAAAACTAGCCAGGCGTGATGGCATGCACCTGTAATC
CCAACTACTTAGGAGGCTGAGGCAGGAGAATCGCTTCAACTCGGGAGGCAGAAG
TTGCAGTGAGCCAAGATTGCACCATTGCACTCCAGCCTGGGCAACAAGAGCAAA
ACTACGTCTCAAAAAATAATAATAACAATAAAATAAAAAACAAGCTTTTTTTTTTT
GAAACAGGATCTCACTCCATCACCCAGGCTGGAGTGCAGTGGCACGATCTTGGCT
CACTGCAACCTCCGCCTCCCGGGTTCAAGTGATTCTCATGCCTCGGCCTCCTGAGT
AGCTGAGACCACAGGCGCATGCCACCACACCTGGCTAATTTAGAATAAAAAAGAA
GCTTCCTCTCTGCCACTCAGGTAGCCTTATCCCTAATCTCAGCCTCCGTCAGGGAC
TCCCTGAGGCCAGTTGGCTGAAAGCTGCCCAGGGAGTTCTAAGGATTTCAGTTTC
TCTTTCCTTCTTGATGCAGCTCCCAGCTCACTTGGCCCTGCCCACACCTGTTCCCT
CATCAGGCTCCCAGACGGGCCCCGCCCACTCATGCCTCTTAAGTCAAAGTGGAAA
TTCTCATTTCCAATTACCTTTTCACTTTACACACATCATCTTGGATTGCCCATTTTGC
GTGGCTAAAAAGCAGAGCCATGCCGCTGGGGAAGCAGTTGCGATTTAGCCATGGC
TGCAGCTTGGACCGTGGTGCTGGTGACTTTGGTGCTAGGCTTGGCCGTGGCAGGC
CCTGTCCCCACTTCC

Primers:
pGL3-IL29-KpnI-F: GG*GGTACC*ACCATGCCCAGCTGCACATCCC
pGL3-IL29-XhoI-R: CCG*CTCGAG*CAAAGTCACCAGCACCACGGTCC
